# LY6/PLAUR domain containing 3 (LYPD3) maintains melanoma cell stemness and mediates an immunosuppressive microenvironment

**DOI:** 10.1186/s13062-023-00424-3

**Published:** 2023-11-03

**Authors:** Yi-dou Hu, Ke Wu, Yuan-jie Liu, Qian Zhang, Hui Shen, Jin Ji, Dong Fang, Song-yang Xi

**Affiliations:** 1 Zhangjiagang TCM Hospital Affiliated to Nanjing University of Chinese Medicine, Zhangjiagang, 215600 Jiangsu People’s Republic of China; 2 The Second Affiliated Hospital of Soochow University, Suzhou, 215004 Jiangsu People’s Republic of China; 3 No. 1 Clinical Medical College, Nanjing University of Chinese Medicine, Nanjing, 210023 Jiangsu People’s Republic of China; 4 Nanjing University of Chinese Medicine, Jiangsu Province Hospital of Chinese Medicine, Nanjing, 210029 Jiangsu People’s Republic of China; 5 Zhenjiang Hospital of Chinese Traditional and Western Medicine, Zhenjiang, Jiangsu 212000 People’s Republic of China

**Keywords:** Melanoma, Catenin, Cancer stem cell, *LYPD3*, Immunotherapy, Glycolysis

## Abstract

**Background:**

Malignant melanoma is a highly heterogeneous skin cancer with the highest mortality rate among dermatological cancers. Catenins form functional networks in the nucleus to regulate gene expression and determine cell fate. Dysregulation of catenin expression correlates with the malignant characteristics of the tumor. We aimed to investigate the regulatory mechanisms of catenins in melanoma and to further define the function of catenin-related molecular signaling in the tumor microenvironment.

**Methods:**

In this study, a bioinformatics approach combined with experimental validation was used to explore the potential tumor biology mechanisms of catenin-related signaling.

**Results:**

Melanoma patients can be divided into two catenin clusters. Patients defined by high Junction Plakoglobin (*JUP*), Plakophilin 1 (*PKP1*), Plakophilin 3 (*PKP3*) levels (C2) had shorter survival time than other patients (C1). We demonstrated that JUP regulates Anterior Gradient 2 (AGR2)/LY6/PLAUR Domain Containing 3 (LYPD3) to maintain melanoma stemness and promotes glycolysis. We also found that LYPD3 was co-expressed with S100A9 and associated with immunosuppressive tumor microenvironment (TME).

**Conclusion:**

The JUP/AGR2/LYPD3 signaling axis plays an important role in the malignant features of melanoma. Targeting the JUP/AGR2/LYPD3 signaling axis can help develop promising drugs.

**Supplementary Information:**

The online version contains supplementary material available at 10.1186/s13062-023-00424-3.

## Introduction

As one of the most aggressive cutaneous malignancies, melanoma poses a major public health challenge and its incidence has been increasing over the past decade [1, 2]. Although, due to prominent immunogens, significant breakthroughs have been made in melanoma immunotherapy, particularly with the use of immune checkpoint Immune checkpoint blockades (ICBs) [3–5]. Unfortunately, there is still a significant percentage of patients who struggle to benefit from ICBs and the frequent band of anti-Programmed Cell Death-1 (PD-1) drugs brings intolerable adverse effects [6]. Considering that the mechanism of tumor response to ICBs is regulated by multiple factors, it is necessary to further elucidate it at the molecular level [7–9]. Many studies have confirmed that the tumor microenvironment (TME), on which tumor cell survival depends, is highly heterogeneous and that multiple pathways exist to suppress anti-tumor immunity [10–12]. For example, tumor-infiltrating lymphocytes (TILs) with low abundance or dysfunctional tumors do not respond significantly to ICBs [13, 14].

Most vertebrates have 12 different types of gene encoding catenins that belong to four subfamilies, including alpha catenins, beta catenins, delta catenins, and p120 catenins [15]. Among them, β-catenins have been widely demonstrated to be targets of Wnt signaling [16], specifically, the degradation of cytoplasmic β-catenins upon binding of Wnt to its receptor, thereby facilitating its nuclear translocation [17]. Interestingly, nuclear β-catenins often co-exist with PD-1, and it has been shown that β-catenins promote immune escape by impairing T cell activity through defective recruitment of dendritic cells [18]. In addition to the classical β-catenins signaling pattern, other linker proteins have equally promising emerging functions [19–21]. Since all catenins possess a central armadillo domain that is well suited for nucleo-cytoplasmic shuttling, it is easy to understand that they play important and complex roles in the organism [22–24]. Indeed, the expression of many genes is regulated by multiple catenins that act synergistically or antagonistically [25]. Importantly, there is a frequent shared relationship between catenins and actin, with α-catenin binding to filamentous actin (F-actin) and thereby mediating the attachment of the cadherin-β catenins complex to the cytoskeleton [26, 27]. This reflects the importance of catenins in cell development and differentiation. However, the commonalities and differences in the functions of catenins and the extent to which they network or act independently remain largely unknown.

Cancer stem cells (CSCs) are subpopulations of cells in tumors that are in a stem cell state and have stem cell characteristics, which partly explain the heterogeneity of tumors [28]. It is well documented that CSCs are an important cause of treatment resistance, metastatic recurrence, and immunosuppression in tumor patients [29]. As an intrinsic tumorigenic mechanism, catenin signaling is associated with cancer Epithelial mesenchymal transition (EMT) and stem cell-like biological phenotypes [30, 31]. A previous study demonstrated that β-catenin-positive tumors were unresponsive to immune checkpoint therapy compared to murine tumors lacking β-catenin [32]. These studies suggest a potential link among catenins, stem cell-like phenotypes, and immunosuppression.

In this study, we analyzed the gene expression and transcriptional heterogeneity of 12 catenins in 32 cancer types and assessed their prognostic value. The results suggest that genomic changes, including DNA methylation and Copy number variation (CNV), affect the transcriptional levels of catenins. We focused on the regulatory mechanisms of catenins in melanoma. Linking catenins to stem cell-like features of melanoma cells and immunosuppressive TME was done by The Cancer Genome Atlas (TCGA) and Gene Expression Omnibus (GEO) bulk data. Our results suggest the presence of Plakoglobin (JUP)/Anterior Gradient 2 (AGR2)/LY6/PLAUR Domain Containing 3 (LYPD3) pro-oncogenic signaling in melanoma. We also found that LYPD3 was closely associated with S100 Calcium Binding Protein A9 (S100A9)-labelled myeloid cells, impairing immunotherapy response. We confirmed that the JUP/AGR2/LYPD3 signaling axis promotes F-actin expression, remodels the cytoskeleton and confers a robust invasive phenotype to melanoma cells.

## Materials and methods

All specific bioinformatics methods and experimental approaches are described in the Supplementary Methods.

### Antibodies, reagents, and cell lines

The details for the wet-lab experiments and all antibodies, reagents, and cell lines are summarized in Additional file 1: Table S1–S5.

### Data source and process

RNA-seq transcriptome information and associated clinical data for 471 patients with skin cutaneous melanoma (SKCM) were downloaded from the TCGA portal, while a portion of the TCGA data was processed using the Gene Set Cancer Analysis (GSCA) online tool. And, additional independent validation cohorts (GSE22153, GSE59455, and GSE65904) were obtained from the Gene Expression Omnibus (GEO) database [33, 34]. In addition, two immunotherapy datasets (Nathanson_2017 and GSE120575) were processed through the Tumor Immunotherapy Gene Expression Resource (TIGER) online tool [35]. All gene expression data were exported as a standardised data matrix with the help of R software.

### Statistical analysis

We used Spearman and Pearson correlation coefficients to determine the correlation between variables. The Mann–Whitney U-test (also known as the Wilcoxon rank sum test) was used to analyze non-normally distributed variables. One-way analysis of variance (ANOVA) test was used to compare two or more groups. The log-rank (Mantel-Cox) test was used for survival analysis. The univariate Cox regression was utilized to identify factors with independent prognostic value, and the corresponding hazard ratios (HR) were provided. Statistical analyses were performed using the programming language software R and Excel (Microsoft) and a P value (two-tailed) < 0.05 was considered statistically significant.

## Results

### Genetic characterization, transcriptional variation and expression patterns of 12 catenin molecules

**Fig. 1:**
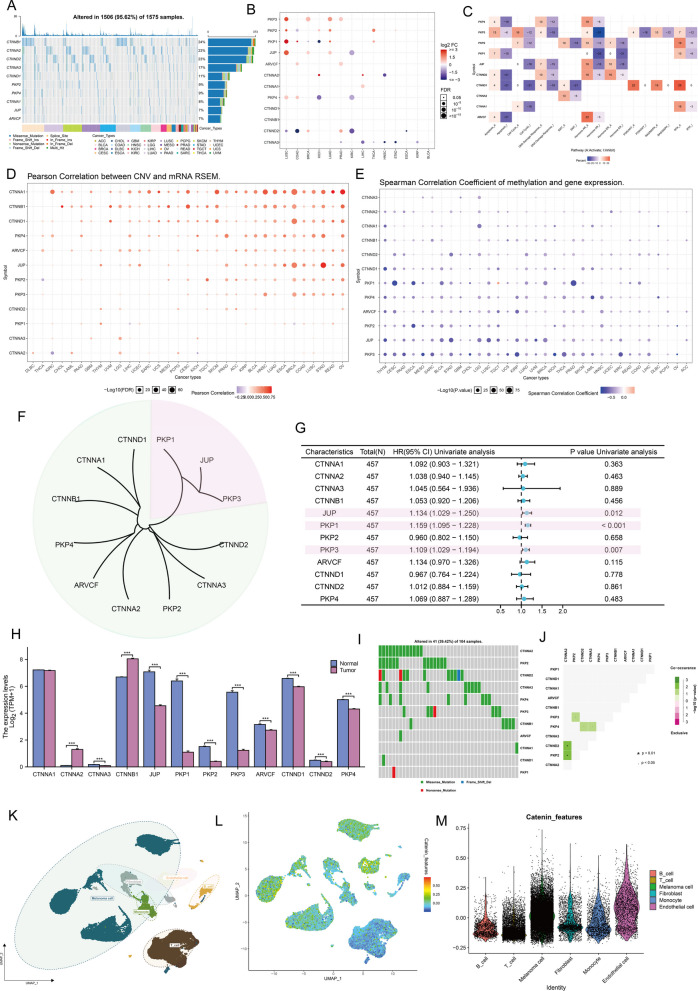
Expression variation of catenin molecules. **A** The waterfall plot shows somatic mutations in the 10 catenins with the highest mutation frequency in the pan-cancer analysis. 95.62% is the percentage of 1575 cancer samples with 1506 mutations in at least 10 genes. The percentage plot (right side of the figure) is the number of samples with the corresponding gene mutation divided by 1575 samples with at least one mutation among the 10 catenins coding genes. **B** The color of these dots represents the degree of variance. Red dots represent high expression in cancerous tissues, blue dots represent the opposite. Fold change equals mean (tumor)/mean (normal), a t-test was used, and p values were False-discovery rate (FDR) corrected. The size of the bubble indicates the FDR. The larger the bubble, the lower the FDR. Genes with fold change (Fold change > 2) and significance (FDR > 0.05) were retained to generate plots. If there are no significant genes in a cancer type, omit that cancer type from the final graph. **C** The heatmap shows the correlation between the expression levels of 10 catenins and important cancer-related signaling pathways. The percentage of cancers in which each catenin-encoding gene has an effect on this pathway is shown for each of the 32 cancer types: (number of activated or repressed cancer types/total number*100%). Catenin-encoding genes that are functional (inhibited or activated) in at least 5 cancer types are shown. “Pathway activation” (red) indicates the percentage of cancers in which a pathway may be activated by a given gene, and inhibition is shown in a similar way as “pathway inhibition” (blue). **D** The bubble plots show the correlation between Copy number variation (CNV) and mRNA expression levels. Red color indicates positive correlation and blue color indicates negative correlation. Darker colors indicate larger correlation coefficients. The size of the bubbles represents the FDR. **E** The bubble plot shows the correlation between methylation of 10 catenin molecules and mRNA expression. Red color indicates positive correlation and blue color indicates negative correlation. Darker colors indicate larger correlation coefficients. The size of the bubbles represents FDR. **F** Hierarchical clustering dendrogram showing the Euclidean distance between genes calculated using the “dist” function. The 2 major clusters identified are indicated by green and pink boxes, respectively. **G** The forest plot shows the results of Cox regression analysis of overall survival (OS) for 10 catenin molecules in the The Cancer Genome Atlas (TCGA)- Skin cutaneous melanoma (SKCM) cohort. **H** mRNA level of catenin coding genes between cancer tissues in the TCGA-SKCM cohort (n = 469) and control tissues (n = 812). Normal tissues in both the TCGA and GTEx databases were included as negative controls. ***P < 0.001 **I** Mutation frequency of 10 catenin molecules in 104 cutaneous melanoma patients in the TCGA-SKCM cohort. **J** Mutational characterization of 10 catenin molecules in 104 patients with cutaneous melanoma in the TCGA-SKCM cohort; green indicates co-mutations and asterisks indicate P values (*P* < 0.05, *P < 0.01). **K** Cells were clustered into 6 types via Uniform Manifold Approximation and Projection (UMAP) plot dimensionality reduction algorithm. Different cell types are represented by different colors. **L**–**M** UMAP (**L**) and Violin (**M**) plots visualization of the catenin feature (obtained based on the “AddModuleScore” function in “Seurat” package)

Information about the 12 catenins was downloaded from the Human Genome organization gene nomenclature committee (HGNC) portal. The 12 molecules are Catenin Alpha 1 (*CTNNA1*), Catenin Alpha 2 (*CTNNA2*), Catenin Alpha 3 (*CTNNA3*), Catenin Beta 1 (*CTNNB1*), *JUP*, Plakophilin 1 (*PKP1*), Plakophilin 2 (*PKP2*), Plakophilin 2 (*PKP3*), ARVCF Delta Catenin Family Member (*ARVCF*), Catenin Delta 1 (*CTNND1*), Catenin Delta 1 (*CTNND2*), and Plakophilin 4 (*PKP4*). To examine genetic variation in catenin molecules in cancer, we selected 1575 samples with at least one mutation from 12 catenin molecules in the TCGA pan-cancer database (Fig. 1A). Considering that only 14 cancer types had more than 10 paired tumor and normal samples, we provided differential mRNA expression of catenins in these 14 cancer types. The expression of different catenin molecules in different cancer tissues was inconsistent, and several molecules, including *PKP3*, *PKP2*, *PKP1*, and *JUP*, were overexpressed in several cancer tissues (Fig. 1B). We also explored the characteristics of catenin-related signaling pathways. At the pan-cancer level, most signaling pathways, particularly *PKP4*, *PKP2*, *JUP*, and *CTNND2*, showed high levels of activation in the Hormone AR signaling pathway, but consistent inhibition of apoptosis, cell cycle, and DNA damage responses (Fig. 1C). Importantly, we demonstrated that catenin expression may correlate with genetic variation. expression levels of CNV and mRNA were positively correlated in most cancer types, particularly for *CNTTA1*, *CNTTB1*, and *CNTTD1* (Fig. 1D). In contrast, in most cancers, gene methylation levels were negatively correlated with mRNA expression levels (Fig. 1E). This part of the analysis showed that catenin expression patterns were highly heterogeneous across cancers and that its aberrant expression was associated with genomic variation.

The focus of this study is on malignant melanoma. We first performed a hierarchical clustering of 12 catenins based on the euclidean distance matrix in TCGA-SKCM and found that *PKP1*, *JUP*, *PKP3* (pink) had a significantly different expression pattern from other catenins (green) (Fig. 1F). Further COX regression analysis indicated that *PKP1*, *JUP*, *PKP3* were significant detrimental factors for Overall survival (OS) in SKCM patients (Fig. 1G). As there was only one normal skin sample in the TCGA-SKCM data, we included normal tissue from the Genotype Tissue Expression (GTEx) database as a negative control to measure the difference in catenins expression between diseased and normal tissue. As shown in Fig. 1H, aside from *CTNNA1*, *CTNNA2*, and *CTNNB1*, there was low expression of most catenins in the tumor samples (*P* < 0.001) compared with the controls. On the other hand, catenin molecular mutations occurred in 41 of the 104 samples, with a mutation frequency of 39.13%. Among the 104 cases, *CTNNA2* had the highest mutation frequency, all of which were missense mutations (Fig. 1I). In TCGA-SKCM, there were significant co-mutations between *CTNNA2* and *CTNND2*, *CTNNA2* and *PKP2*, *PKP2* and *PKP3*, *CTNND2* and *PKP4*, and *CTNNA3* and *PKP4* (*P* < 0.05, Fig. 1J). Finally, to better understand the expression patterns of catenins in TME, we utilized a melanoma single-cell dataset (Additional file 1: Fig. S1). Annotation of cells by unique gene markers for each cell type revealed that catenins were expressed at low levels in immune cells, including T cells, B cells, and monocytes (Fig. 1K–M). Results strongly suggested that the expression of catenins was highly heterogeneous and associated with tumorigenesis.

### Two different catenin expression patterns identified by unsupervised learning

**Fig. 2:**
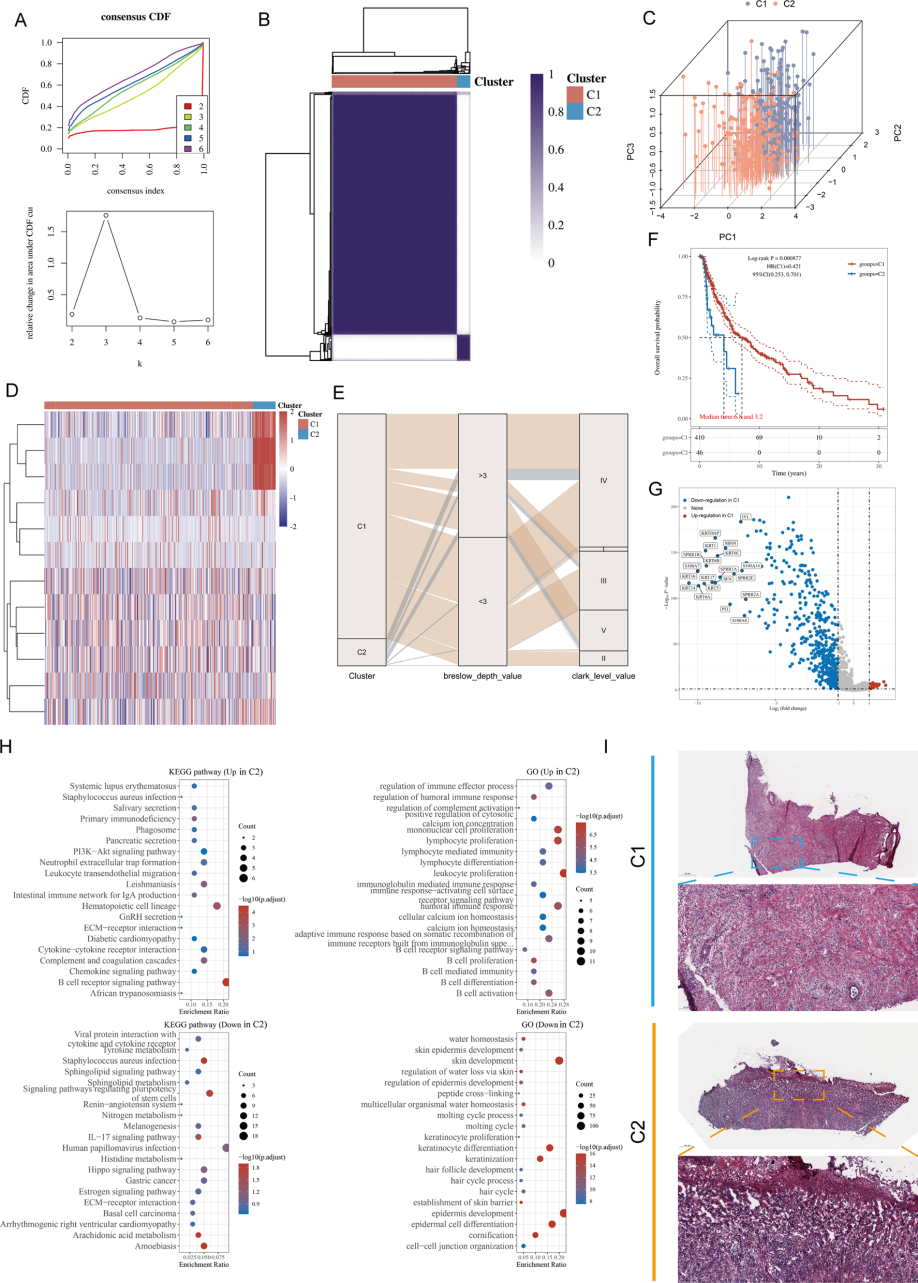
Unsupervised clustering to identify two catenin classifications. **A** Unsupervised Machine Learning algorithms was used to identify 2 molecular subtypes in TCGA-SKCM. **A** Left: The cumulative distribution function (CDF) curves in consensus cluster analysis. CDF curves of consensus scores by different subtype numbers (k = 2, 3, 4, 5, and 6) were displayed. Right: Relative change in area under the CDF curve for k = 2–6. **B** The consensus score matrix of melanoma cases in TCGA-SKCM when k = 2. The higher the consensus score was, the more likely they were assigned to the same group. **C** Three-dimensional principal component analysis (PCA) plot showing the distribution of melanoma examples based on catenin levels when k = 2. Each point represents a sample and different sample clusters are marked using different colors. **D** Heatmap showing differences in expression of 10 catenin molecules between two melanoma clusters. **E** The Sankey diagram demonstrated the association between clinicopathological parameters (Breslow_depth, Clark_level, and T/N/M stage) and subtypes attributes. **F** OS analysis to the catenin subtype was performed. Log rank test was conducted. **G** Volcano map of differentially expressed genes (DEGs) between C1 and C2 in TCGA-SKCM dataset. Data on the abscissa are differences in gene expression (log2 fold change); data on the ordinate represent the significance of these differences (− log10 padj). Red indicates upregulation in C1, and blue indicates downregulation in C1. **H** Gene Ontology (GO) and Kyoto encyclopedia of genes and genomes (KEGG) enrichment analysis was performed using the “clusterProfiler” R package. The size of the bubbles represents the number of genes enriched and the color of the bubbles represents the significance level [− log10 (P.adjust)] of the enrichment. **I** Representative pictures of pathologic Hematoxylin and eosin (HE) staining of the two catenin phenotypes

To fully understand the intergratd mechanism of catenins in melanoma, we performed unsupervised clustering of 456 samples from TCGA-SKCM using the R package “ConsensusClusterPlus”. Two unique catenin-modified phenotypes were identified, named cluster 1 (C1, 410 cases), and cluster 2 (C2, 46 cases) (Fig. 2A, B, Additional file 1: Fig. S2). Principal component analysis (PCA) showed that the two clusters had significantly different catenins expression patterns, confirming the validity of the clustering (Fig. 2C). The heat map showed that *PKP1*, *JUP*, and *PKP2* were significantly overexpressed in C2 than in C1 (Fig. 2D). To ensure stability of clustering, the same process was performed in merged GEO (mGEO) data (Additional file 1: Fig. S3). We then compared our phenotype with commonly used clinical staging indicators. In contrast to C1, there are mostly advanced subtypes in C2, particularly a high proportion of Breslow thickness ≥ 3 mm and Clark’s Level IV, and V tumor (Fig. 2E). Survival analysis showed that C1 provided a particularly significant survival advantage, whereas C2 had a poorer prognosis (log-rank, *P* = 0.000087, Fig. 2F). We further examined the 10 genes with the highest mutation frequencies in each subgroup (Additional file 1: Fig. S4). Interestingly, *BRAF* had a high mutation frequency in C2, and *BRAF* mutations have been shown to be a driver of poor prognosis in melanoma patients. The mutation profiles of genes frequently mutated in C1 and C2 are shown in oncoplots.

We next analyzed the differentially expressed genes (DEGs) between different catenin subtypes and explored the characterization of the associated molecular signals. Based on the “limma” R package, we obtained 511 genes highly expressed in C2 and 40 genes highly expressed in C1 (Fig. 2G). We then performed Gene Ontology (GO) and Kyoto encyclopedia of genes and genomes (KEGG) enrichment analyses based on DEGs (Fig. 2H). In KEGG terms, we observed that the B-cell receptor signaling pathway and hematopoietic cell lineage were down-regulated in C2, while the IL-17 signaling pathway, gastric cancer, and basal cell carcinoma were up-regulated in C2. In GO terms, immune system-related signals were downregulated in C2, whereas skin development and keratinocyte differentiation signals were upregulated in C2. Notably, histopathological sections confirmed that tumor cells in C2 showed more severe cytological heterogeneity compared to C1, suggesting a more significant degree of malignancy (Fig. 2I).

### Identification of LYPD3 as one of the underlying regulators of catenin phenotype

**Fig. 3:**
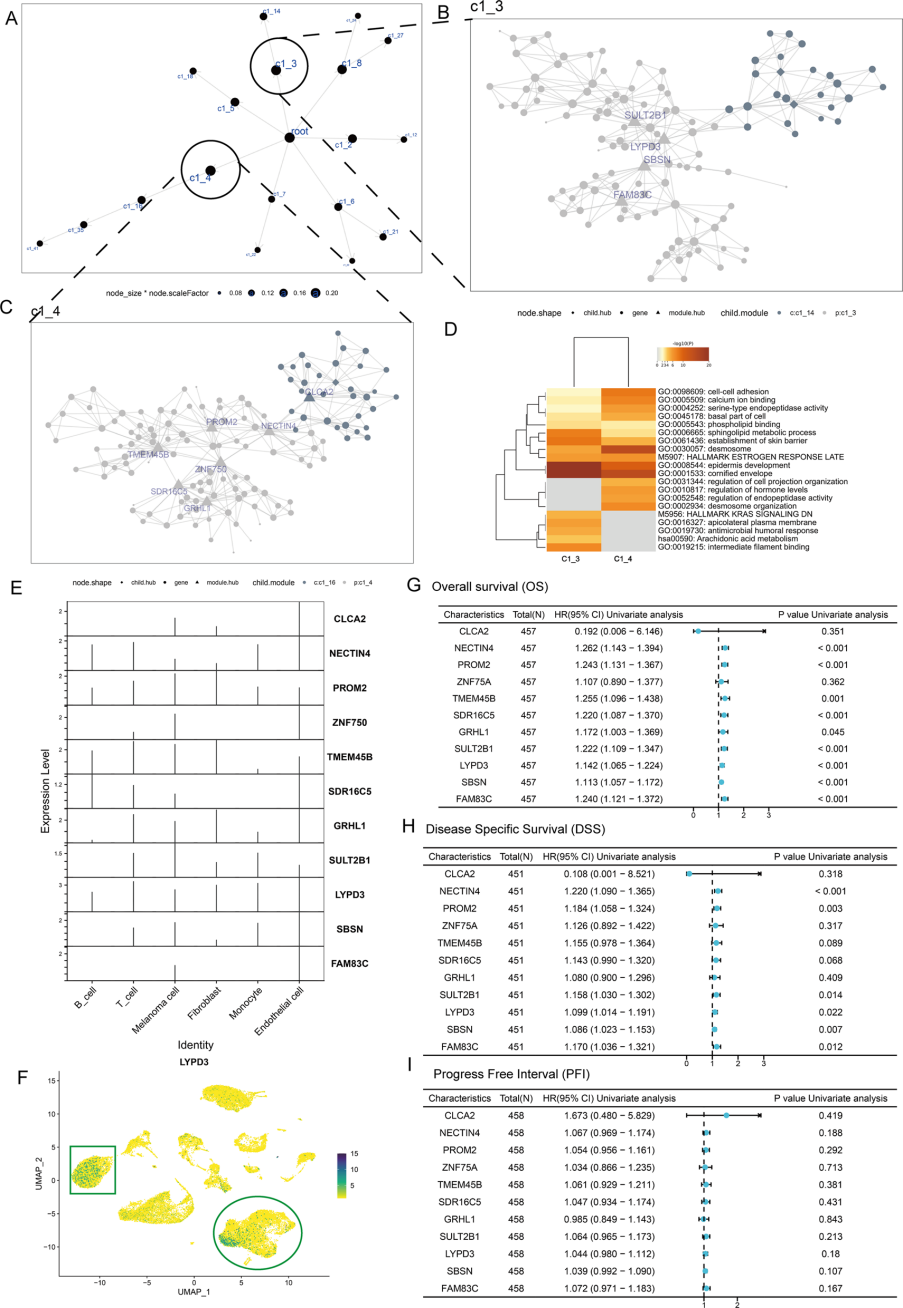
Bulk data combined with single-cell data to identify *LYPD3* as a study subject. **A** Co-expression network constructed based on DEGs between C1 and C2. **B**, **C** The Multiscale Embedded Gene co-expression Network Analysis (MEGENA) network shows the two largest gene modules. Different colors represent genes in different modules, and triangles represent hub genes in the modules. **D** GO and KEGG functional enrichment analysis demonstrated shared and unique biological signals of C1_3 and C1_4. **E** Violin plots show the expression of hub genes in C1_3 and C1_4 across 6 cell types in GSE189889. **F** UMAP plot showing the expression levels of LYPD3, defined for six cell types. **G–I** The forest map shows the results of Cox regression analysis on the OS (**G**), Disease Specific survival (DSS, H), and Progrssion Fre Interval (PFI, I) of 10 hub genes in the TCGA-SKCM cohort

To identify potential regulators in the catenin phenotype, the DEGs identified above were applied to a Multiscale Embedded Gene co-expression Network Analysis (MEGENA) (Fig. 3A). We identified 18 modules and 551 module genes. The largest module, C1_4, consisted of 142 genes, followed by C1_3 with 122 genes. Figure 3B, C demonstrated the network structure of C1_3 and C1_4. In addition, Metascape enrichment analysis showed that C1_3 and C1_4 exhibited consistent correlations with regulating epidermal development and cornified envelope (Fig. 3D).

To further understand the significance of C1_3 and C1_4 in TME, we extracted the hub genes in the module and demonstrated their expression levels by violin plots (Fig. 3E). Comparison of hub gene expression levels in different cell types was established and showed that *LYPD3* was expressed in the highest abundance and upregulated in both melanoma cells and immune cells (Fig. 3F, green circle and box). Finally, associations between hub genes and OS, progression-free interval (PFI), and disease-specific survival (DSS) were modelled using Cox proportional risk regression (Fig. 3G–I). The results showed that, Nectin Cell Adhesion Molecule 4 (*NECTIN4*), Prominin 2 (*PROM2*), Transmembrane Protein 45B (*TMEM45B*), Short Chain Dehydrogenase/Reductase Family 16C Member 5 (*SDR16C5*), Grainyhead Like Transcription Factor 1 (*GRHL1*), Sulfotransferase Family 2B Member 1 (*SULT2B1*), *LYPD3*, Suprabasin (*SBSN*), and Family With Sequence Similarity 83 Member C (*FAM83C*) were significant unfavorable factors for OS, and *NECTIN4*, *PROM2*, *SULT2B1*, *LYPD3*, *SBSN*, and *FAM83C* were significant unfavorable factors for DSS (*P* < 0.05). Thus, we focus on *LYPD3* in the next section.

### Identification of the JUP/LYPD3/AGR2 signaling axis on melanoma cells

**Fig. 4:**
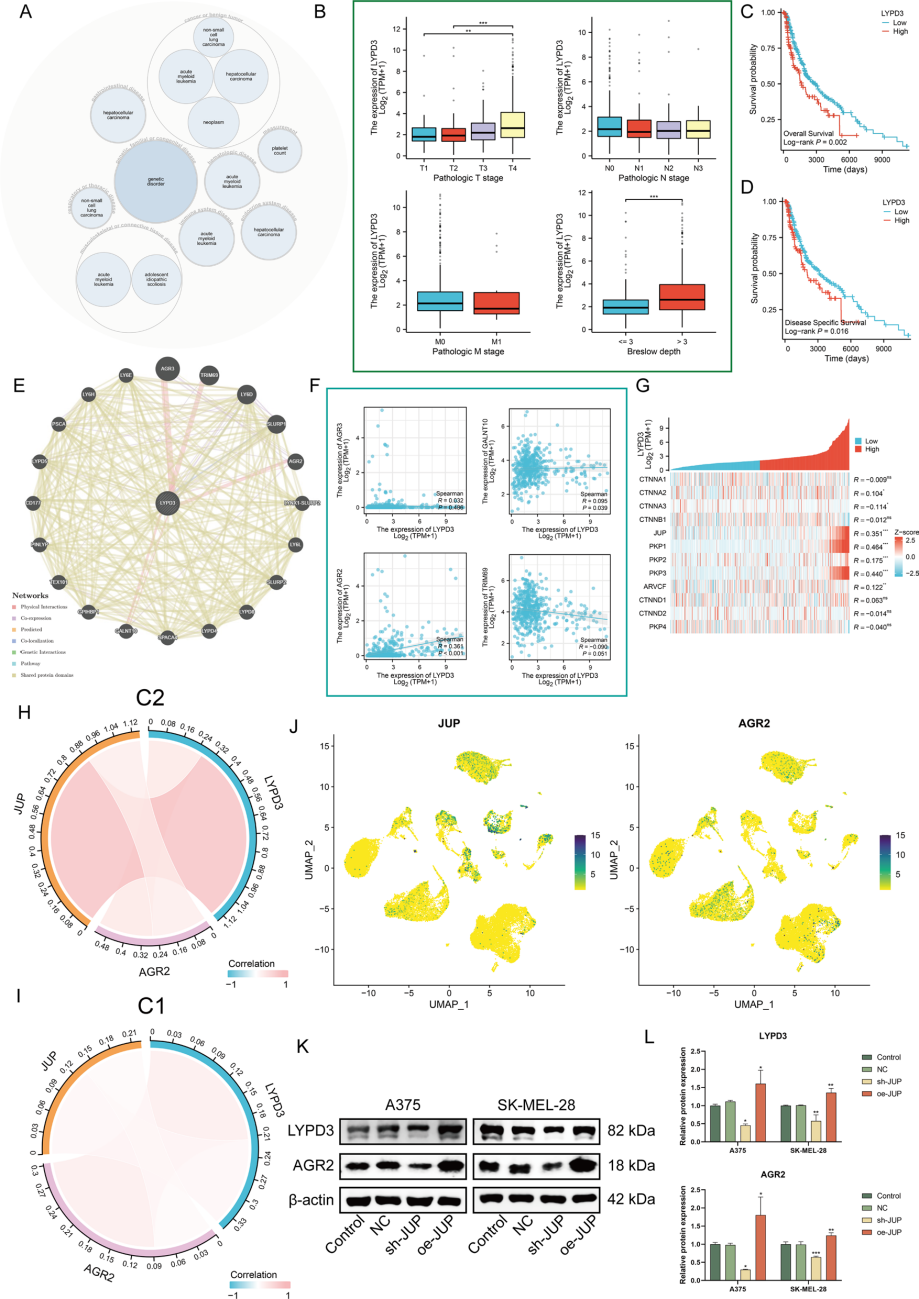
Bioinformatics combined with in vitro experiments to determine the presence of JUP/AGR2/LYPD3 signaling in melanoma cells. **A** Pathway and disease analysis of *LYPD3*. **B** Boxplot showing the expression level of *LYPD3* among different pathologic stages (stage T, N, M and Breslow depth). **C**, **D** OS (**C**) and DSS (**D**) analysis according to the expression level of the *LYPD3* gene were performed using melanoma cases in the TCGA-SKCM cohort. **E** The gene–gene interaction network for *LYPD3* and neighboring genes was analyzed using the GeneMANIA database. Each node represents a gene. The line color represents possible relationship between the respective genes. **F** Correlation between *LYPD3* level and four physically interacting genes as analyzed in TCGA-SKCM. Spearman method was applied. **G** Heatmap showing the correlation between *LYPD3* and ten catenins, and the Spearman correlation coefficients are provided on the right side of the graph. **H**, **I** The positive correlation among *LYPD3*, *JUP*, and AGR2 was stronger in C2 than in C1. **J** UMAP plot demonstrating the expression of *AGR2* and *JUP*. Higher expression is indicated by a greener color. (K-L) Verification of *AGR2* and *LYPD3* expression by Western blotting assay in melanoma cells transfected with oe and sh-JUP. One-way ANOVA was applied. *ns* not significant, *P < 0.05, **P < 0.01, ***P < 0.001

Herein, we investigated LYPD3-associated cancer biology functions. LYPD3 has been shown to be highly expressed in several human malignancies, suggesting a potential oncogenic role [36–38]. Open Targets software showed that *LYPD3* plays an important regulatory role in cancer or benign tumor (Fig. 4A). Figure 4B showed correlation between *LYPD3* expression and clinicopathological characteristics of melanoma patients, indicating that *LYPD3* was positively correlated with several melanoma classifications, namely, T classification for TNM staging (*p* < 0.01), and Breslow depth (*p* < 0.001). The latter is considered the strongest predictor of melanoma mortality, with greater Breslow tumor thickness representing more severe local invasion [39]. Further, the level of *LYPD3* was determined to be negatively correlated with survival (Fig. 4C, D, logrank *P* < 0.05). These results provide solid evidence for *LYPD3* as a key regulator and stimulator of melanoma progression.

Subsequently, we utilized GENEMINIA to build a network of *LYPD3* and its interacting genes. This tool identified four proteins that physically interact with *LYPD3*, including Anterior Gradient 3 (*AGR3*), Tripartite Motif-Containing Protein 69 (*TRIM69*), Anterior Gradient 2 (*AGR2*), and Polypeptide N-Acetylgalactosaminyltransferase 10 (*GALNT10*) (Fig. 4E). We computed transcriptome correlations between *LYDP3* and the four interacting genes in TCGA-SKCM and found a significant positive correlation between *LYPD3* and *AGR2* levels (Fig. 4F, R = 0.361, *P* < 0.001). Interestingly, *LYPD3* showed a significant positive correlation with *JUP* (R = 0.351, *P* < 0.001), *PKP1* (R = 0.464, *P* < 0.001), and *PKP3* (R = 0.440, *P* < 0.001) (Fig. 4G). These three genes were previously found to have similar expression patterns and were negatively associated with prognosis (Fig. 1F, G). It has been demonstrated that LYPD3 is a functional cell surface receptor for AGR2 and that AGR2 is highly regulated by β-catenin signaling [40, 41]. Considering that JUP is a homologue of β-catenin and is closely related to β-catenin in many cases to concertedly regulate cell fate, we speculate that there may be a mechanism of JUP/AGR2/LYPD3 signaling regulation in melanoma. We confirmed that *JUP* and *AGR2* were associated with unfavourable prognosis of melanoma by KM curves (Additional file 1: Fig. S5A, B), and that *AGR2* was associated with higher T stage and deeper Breslow depth (Additional file 1: Fig. S5C, D). Notably, compared to C1, we observed that the correlations among *JUP*, *AGR2*, *LYPD3* was significantly strengthened in C2 (Fig. 4H, I) and that *JUP* and *AGR2* were synchronously expressed on melanocytes (Fig. 4J). To verify our speculation, a stably transfected cell line for *JUP* was established and western blot was performed. As shown, the expression of *JUP* knockdown mediated by the shRNA group significantly reduced AGR2/LYPD3 signaling activity, whereas the opposite was true for oeRNA-mediating *JUP* gene overexpression, in agreement with our prediction (Fig. 4K, L, P < 0.05).

### Upregulation of JUP/AGR2/LYPD3 signaling contributes to the maintenance of melanoma cell stemness and enhanced glycolysis levels

**Fig. 5:**
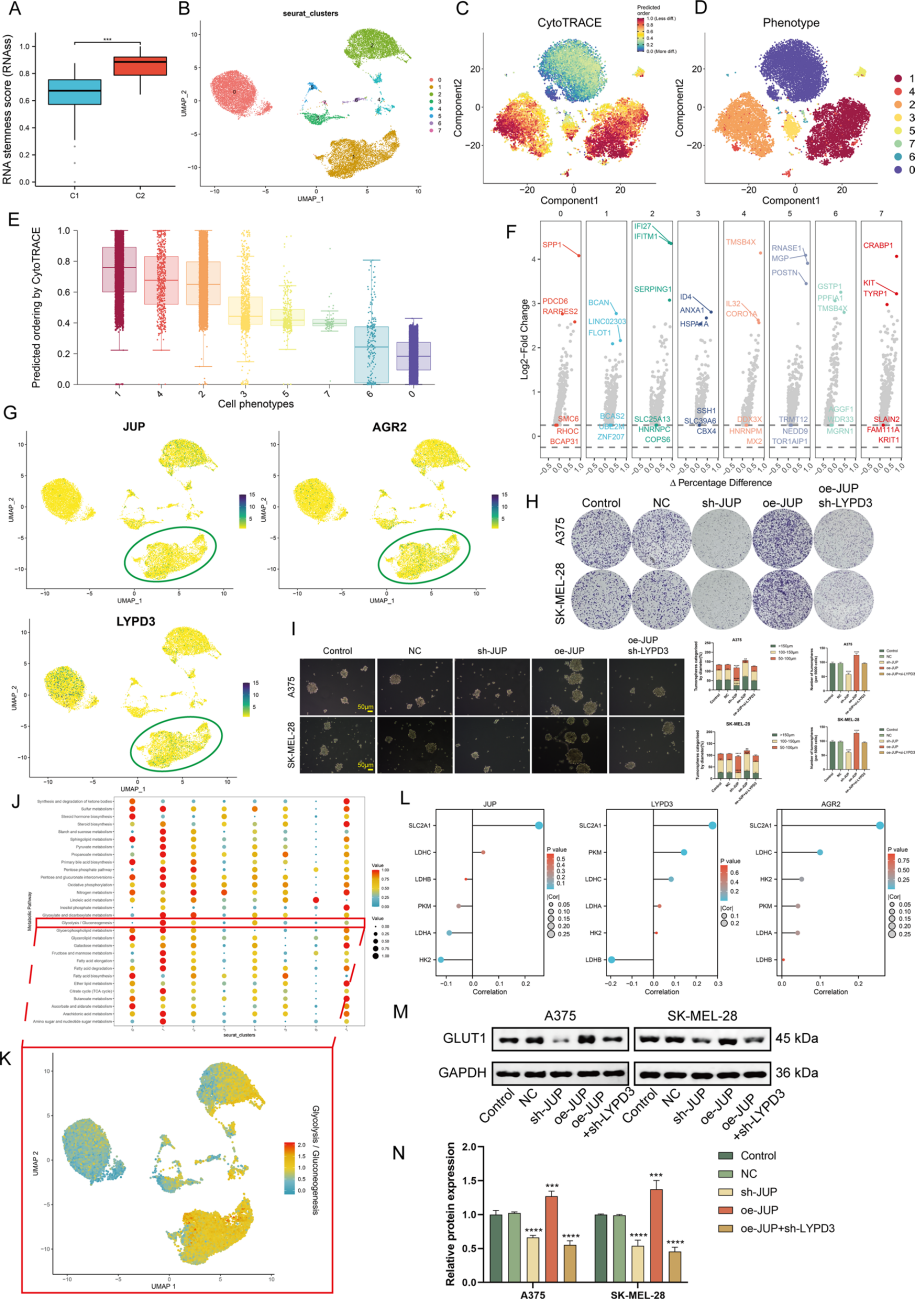
Bioinformatics combined with in vitro experiments to determine that JUP/AGR2/LYPD3 signaling in melanoma cells can maintain stemness. **A** Boxplot showing the difference in RNA stemness score between C1 and C2. **B** UMAP plot of melanoma cells. **C**, **D** tSNE plot demonstrating the degree of differentiation of each melanoma cell cluster assessed by CytoTRACE. **E** Boxplot showing the differentiation score of melanoma cell cluster. **F** Volcano graph demonstrating the feature genes of each melanoma cell cluster. **G** UMAP plot showing the co-localization of *JUP*, *AGR2*, and *LYPD3*. **H** Images of colony formation assay in control, NC, oe-*JUP*, sh-*JUP*, and oe-*JUP* + sh-*LYPD3* melanoma cell group. **I** Images and quantitative analysis of sphere-forming assay in control, NC, oe-*JUP*, sh-*JUP*, and oe-*JUP* + sh-*LYPD3* melanoma cell group. **J**–**K** Bubble and UMAP plots demonstrating upregulation of glycolysis levels in cluster 1 melanoma cells. **L** Lollipop plot demonstrating the Spearman correlation between JUP/AGR2/LYPD3 signaling and five key enzymes of glycolysis in TCGA-SKCM. Only *SLC2A1* (GLUT1) is consistently and significantly correlated with *JUP*, *AGR2*, and *LYPD3*. (M–N) Verification of GLUT1 expression by Western blotting assay in control, NC, oe-*JUP*, sh-*JUP*, and oe-*JUP* + sh-*LYPD3* melanoma cell group. One-way ANOVA was applied. **P < 0.01, ***P < 0.001, ****P < 0.0001

Cell stemness scores based on bulk data showed that C2 exhibited greater stemness (*P* < 0.001, Fig. 5A). Since JUP/AGR2/LYPD3 signaling is more prominent in C2, we speculate that the enhanced stemness phenotype of C2 may be related to this signaling. We performed cluster analysis on 19,547 melanoma cells and identified eight prominent cell subpopulations (Cluster 0-Cluster 7). CytoTRACE analysis indicateed that cluster 1 exhibited significantly less differentiated state than other melanoma cell populations (Fig. 5B–E). Figure 5F demonstrated the highly expressed genes in each cluster, where Brevican (*BCAN*), Long Intergenic Non-Protein Coding RNA 2303 (*LINC02303*), Flotillin 1 (*FLOT1*) were upregulated in cluster 1. We noted that *JUP*, *AGR2*, and *LYPD3* were all expressed in cluster 1 (Fig. 5G), suggesting a potential correlation between them and poorly differentiated status. Clone formation experiments showed that significantly more colonies were formed in the oe-*JUP* group compared to the control group, and this effect was attenuated by sh-*LYPD3* (Fig. 5H). In spheroidal cultures, *JUP* silencing leaded to decreased sphere-forming ability, whereas JUP overexpression produced the opposite effect (*P* < 0.01, Fig. 5I).

The Warburg effect is a common metabolic phenotype in tumors, where CSCs are heavily dependent on glycolysis [42]. We performed the scMetabolism tool to assess the metabolic profile of each melanoma cell subpopulation, and the results showed that the metabolic level of cluster 1 was significantly higher than that of the other subpopulations, especially glycolysis (Fig. 5J, K). To determine by what pathway JUP/AGR2/LYPD3 signaling affects metabolism, we calculated the correlation of signaling molecules with six key enzymes of glycolysis based on TCGA-SKCM (Fig. 5L). The results showed that Solute Carrier Family 2 Member 1 (*SLC2A1*, also named *GLUT1*) was significantly positively correlated with all three signaling molecules (*P* < 0.05). Western blot confirmed that GLUT1 is a downstream regulatory target of JUP/AGR2/LYPD3 signaling (Fig. 5M, N). Together, the results suggest that JUP/AGR2/LYPD3 signaling plays a role in maintaining tumor cell stemness and enhances the glycolytic phenotype.

### High-dimension weighted correlation network analysis (hdWGCNA) analysis highlights the biological properties of cluster 1

**Fig. 6:**
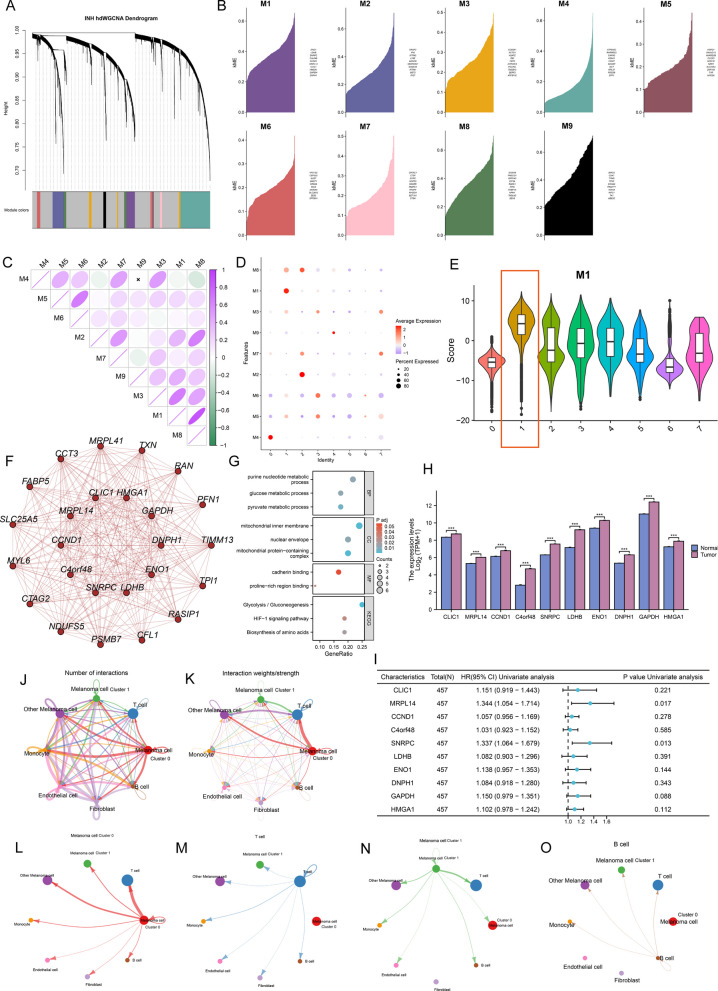
High-dimension Weighted Correlation Network Analysis (hdWGCNA) of melanoma cell reveals the crucial roles of C1. **A** Dendrogram demonstrating nine gene modules in the scale-free network. **B** Nine gene modules and the associated top 10 hub gene were displayed according to the hdWGCNA pipeline. **C** Correlation between nine gene modules. Green represents negative correlation and purple represents positive correlation. **D** Nine module levels in different melanoma cell clusters. **E** Violin plot demonstrating that Module 1 (M1) activity is significantly higher in Cluster 1 than in the other melanoma cell clusters. **F** Protein–protein interaction (PPI) network for the top 25 genes in M1. **G** GO and KEGG enrichmnt analysis of M1. **H** Expression level of M1 hub genes between melanoma tissues in the TCGA-SKCM cohort (n = 469) and control tissues in in both the TCGA and GTEx databases (n = 813). Wilcoxon test, ***P < 0.05. **I** Forest plot showing the results of Cox regression analysis of the mean survival (overall survival) of the 10 M1 hub genes in the TCGA-SKCM cohort. **J**–**K** All cell types were analyzed by Cellchat and melanoma cells were classified according to stemness level as cluster 1 (high), cluster 0 (low), and other melanoma cells (medium). The results showed both interaction numbers and interaction strengths. **L**–**O** Cellchat analysis showed weaker communication strength between melanoma cell cluster 1 and T cells compared to melanoma cell cluster 0

Herein, hdWGCNA pipeline was used to explore the potential features of cluster 1. As shown in Fig. 6A, B, nine gene modules (M1-M9) were obtained, with the top hub gene distributed along the hdWGCNA pipeline. Figure 6C illustrates the correlation between modules. We focused on modules significantly associated with cluster 1 and found that M1 is highly expressed on cluster 1 (red box, Fig. 6D, E, Additional file 1: Fig. S6). Enrichment analysis showed that M1 is involved in a large number of cancer-associated signaling pathways, including glycolysis, cadherin binding, and hypoxia-inducible factor (HIF)-1 signaling (Fig. 6F–G). Further analysis showed that all hub genes in M1 were overexpressed in tumor tissues and were detrimental to OS (Fig. 6H, I, HR > 1). Parallelly, the cell–cell communication analysis was also performed using cellchat tool (Fig. 6J, K). Interestingly, compared to the subpopulation of highly differentiated cells (cluster 0), the subpopulation of poorly differentiated cells (cluster 1) had a relatively weak overall ability to communicate with T cells (Fig. 6L–O). This implies that cluster 1 may contribute to the immunosuppressive state of TME.

### JUP/AGR2/LYPD3 signaling promotes tumor growth and remodels the cytoskeleton

**Fig. 7:**
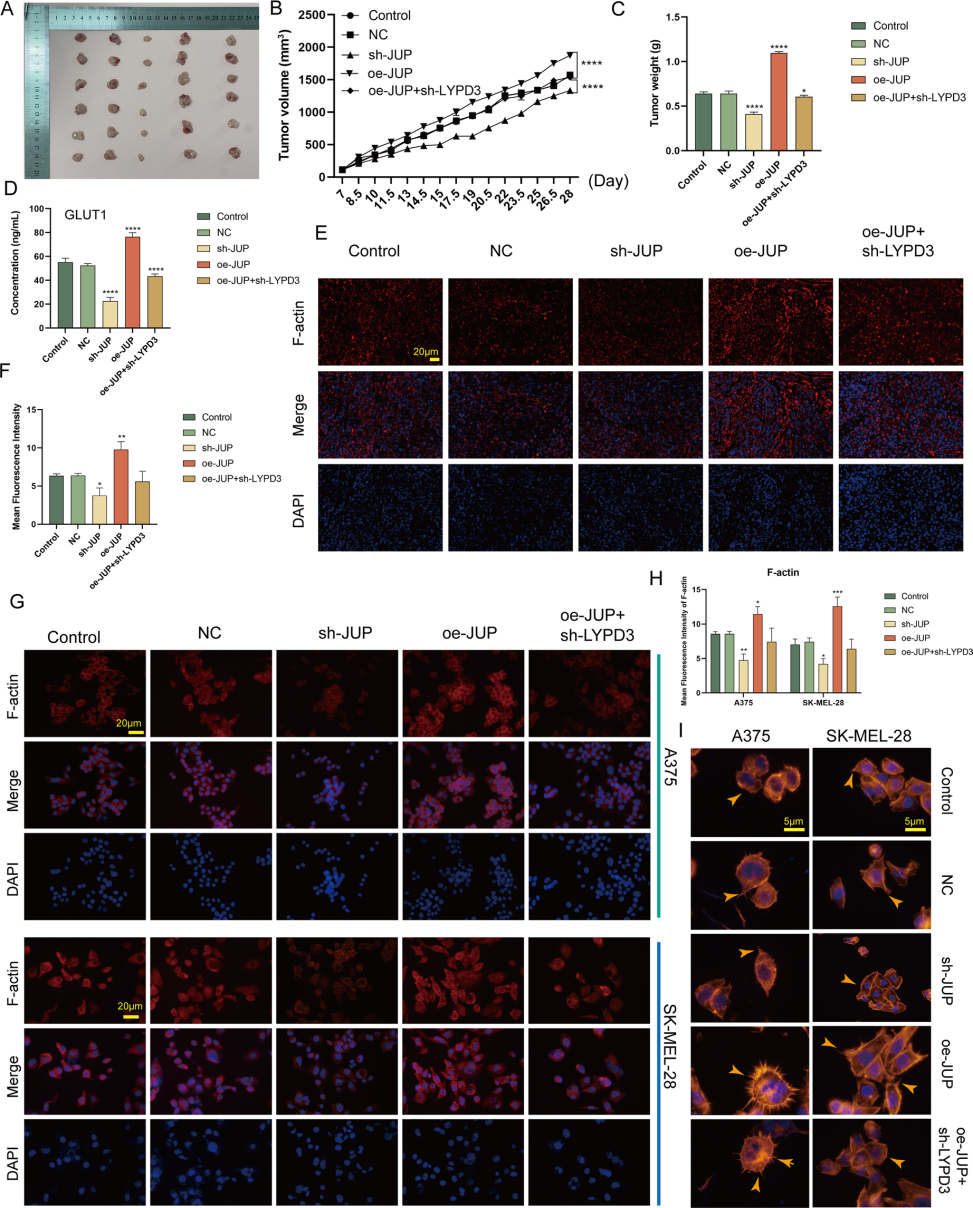
JUP/AGR2/LYPD3 signaling on melanoma cells promotes tumor growth and controls cytoskeletal remodeling. **A**–**C** In vivo experiments showed that overexpression of *JUP* significantly promoted tumor growth and that this effect could be attenuated by sh-*LYPD3*. **D** JUP/AGR2/LYPD3 signaling significantly promoted the expression of the key glycolytic enzyme GLUT1 in vivo. **E**–**H** JUP/AGR2/LYPD3 signaling significantly promoted the expression of the cytoskeletal protein F-actin in vivo (**E**, **F**) and had similar effects in vitro (**G**, **H**). One way ANOVA was conducted. **I** Phalloidin staining showed that JUP/AGR2/LYPD3 signaling remodelled the cytoskeleton and generated more pseudopod structures (yellow arrows). *P < 0.05, **P < 0.01, ***P < 0.001, ****P < 0.0001

We first demonstrated in vivo that upregulation of JUP levels significantly increased tumor growth rate and weight compared to controls, and that this effect was partially reversed by sh-LYPD3 (Fig. 7A–C, P < 0.05). In addition, JUP/AGR2/LYPD3 signaling was shown to have a regulatory role for GLUT1 in vivo (Fig. 7D, P < 0.0001).

Alterations in gene transcription and biological function of cells are often accompanied by morphological changes. It has been established that CSCs drive tumor progression by promoting an invasive phenotype [43]. Furthermore, the highly dynamic and polarized actin cytoskeleton is required for tumor cell migration and invasion into healthy tissue [44]. We demonstrated in vivo that upregulation of JUP/AGR2/LYPD3 signaling contributes to the regulation of the actin cytoskeleton (Fig. 7E, F, P < 0.05) and obtained similar results in in vitro experiments (Fig. 7G, H, P < 0.05). More importantly, cytoskeletal remodeling induced by polymerization of newly generated actin means that cells are able to form specialized cytoarchitectures such as denser filopodia, lamellipodia, and invadopodia. We stained and visualized the cytoskeleton using phalloidin and found that JUP/AGR2/LYPD3 can help melanoma cells form pseudopodia (Fig. 7I).

### Identification of LYPD3 as an immunosuppressive factor

**Fig. 8:**
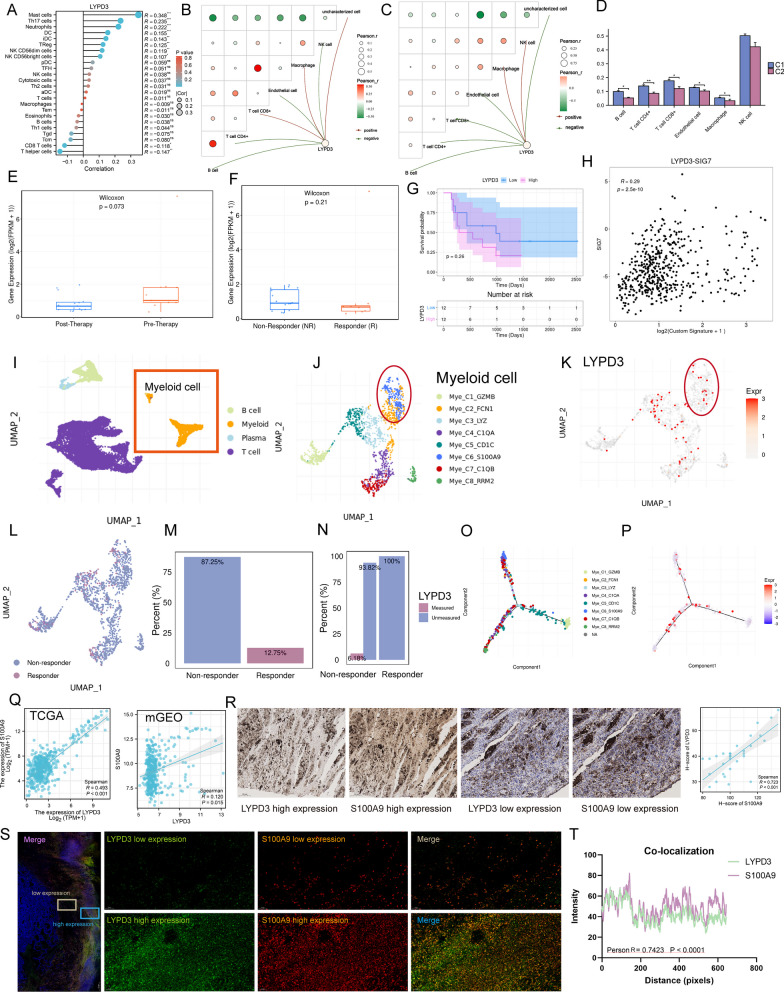
LYPD3 positively correlates with myeloid immunosuppressive marker S100A9. **A** In TCGA-SKCM, *LYPD3* levels were weakly negatively correlated with CD8 + T cell infiltration abundance. **B**, **C** In C2 (**C**), the level of LYPD3 was significantly negatively correlated with the number of CD8 + T cells compared to in C1 (**B**). Green represents negative correlation and red represents positive correlation. **D** Boxplot indicating the infiltration level of immune cells in different catenin phenotypes in the TCGA-SKCM database. Wilcoxon test was conducted. **E** Boxplot showing different *LYPD3* expression between pre-therapy and post-therapy in 24 melanoma patients studied by Tavi Nathanson et al. **F** Boxplot showing different *LYPD3* expression between responder and non-responder after anti-CTLA4 treatment in 24 melanoma patients studied by Tavi Nathanson et al. Wilcoxon test was conducted. **G** Kaplan–Meier plot showing different overall survival outcomes between high and low LYPD3 patient groups in all patients by using data from Tavi Nathanson et al. Logrank test was applied. **H** Correlation between LYPD3 level and T cell dysfunction score in data of Tavi Nathanson et al. Spearman method was used. **I** UMAP plot showing cell types identified by using single-cell RNA-seq dataset GSE120575. **J**–**K** Distribution of LYPD3 expression in different myeloid cell clusters and LYPD3 was co-expressed with S100A9. (L-M) UMAP plot (**L**) and histogram (**M**) showing that myeloid cells were highly enriched in non-responder patients. **N** Histogram showing the proportional expression values of LYPD3 in myeloid cells of responder (0%) and non-responder (6.18%) patients. **O**–**P** Trajectory analysis of myeloid cells and cellular LYPD3 expression profiles in a two-dimensional space. Each point represents an individual cell, coloured by cell type. The solid black line indicates the main diameter path of the minimum spanning tree (MST). **Q** Spearman Correlation between levels of *LYPD3* and *S100A9* in TCGA-SKCM (left) and merged GEO (mGEO, right) cohort. **R** Left: Immunohistochemical (IHC) images of high/low protein expression of LYPD3 and S100A9. Scale bars: 100 µM. Right: Quantifications of LYPD3 and S10A9 IHC staining (H-score) in melanoma cohort (n = 30) showing the positive association between LYPD3 and S100A9 (R = 0.723, *P* < 0.001). **S** The IF staining for LYPD3 and S100A9 in sections derived from melanoma biopsies. Scale bar, 50 µM. **T** The co-localization correlation between LYPD3 and S100A9 was estimated by calculating co-localization coefficients using IamgeJ software. Greater correlation coefficients represent a greater degree of co-localization (Person R = 0.7423, *P* < 0.0001). *P < 0.05, **P < 0.01, ***P < 0.001

As a well-known immunogenic cancer, melanoma is impressive for its abundant antigen and antigen-specific lymphocytes [45]. Therefore, in order to survive and metastasize, melanoma cells must acquire robust immune evasion properties, usually through various pathways mediating T cell dysfunction thereby shaping a broadly immunosuppressive TME [46, 47]. In this study, the “ssGSEA” method was used to estimate immune cell infiltration. We observed a weak negative correlation between *LYPD3* level and CD8 + T cell abundance (Fig. 8A, R = − 0.118, *P* < 0.05). Interestingly, the negative correlation between *LYPD3* and CD8 + T cells was more obvious in C2 compared to C1, and the abundance of immune cell infiltration was lower in C2 (Fig. 8B–D). These data implied that *LYPD3* appeared to correlate with the immunosuppressive profile of C2. Considering that we have demonstrated the regulatory function of *LYPD3* on the maintenance of tumor cell stemness, and that CSCs are usually accompanied by increased expression of immunosuppressive pathways, we speculated that *LYPD3* may be an immunosuppressive factor. To test this conjecture, we included an ICB dataset to examine the relationship between *LYPD3* expression and immunotherapy response. The results showed that *LYPD3* expression was lower in responsive patients than in non-responsive patients and that *LYPD3* levels decreased in patients after ICB treatment (Fig. 8E, F). In addition, patients with high *LYPD3* expression had a poor prognosis (Fig. 8G). Although statistical significance was not reached due to the relatively small sample size, we observed a positive correlation between *LYPD3* levels and T-cell dysfunction score (Fig. 8H, R = 0.29, *P* = 2.5e−10).

To further resolve the immunosuppressive mechanism of *LYPD3*, we examined the relationship between *LYPD3* expression pattern and ICB response at single-cell resolution. As shown in Fig. 8I–K, *LYPD3* was expressed on myeloid cells and T cells. In particular, *LYPD3* was highly expressed on S100A9 + myeloid cells (red box, Mye_C6_S100A9). S100A9 is now thought to be a calcium-binding protein that promotes the accumulation of myeloid-derived suppressor cells (MDSCs) and mediates the formation of malignant loops between MDSCs and CD8 + T cells [48–50]. Further analysis showed that myeloid cells were more abundant in non-responsive patients (87.25%) compared to responder patients (12.75%) and that *LYPD3* was almost exclusively expressed in non-responsive myeloid cells (Fig. 8L–N), reinforcing the evidence that *LYPD3* is a detrimental factor for immunotherapy. Pseudotime analysis showed that *LYPD3* is involved in the differentiation trajectory of myeloid cells (Fig. 8O-P). Finally, we analyzed the correlation between *S100A9* and *LYPD3*. Bulk data showed a significant positive correlation (Fig. 8Q; left, TCGA-SKCM, R = 0.493, *P* < 0.001; right, mGEO, R = 0.120, *P* = 0.015). In addition to the findings at the transcriptional level, Immunohistochemical (IHC) staining confirmed that the protein level of S100A9 increased with increasing LYPD3 protein expression (Fig. 8R, R = 0.723, *P* < 0.001). The co-expression pattern of LYPD3 and S100A9 was confirmed by multiple immunofluorescence (mIF) staining of the same melanoma resection specimen (Fig. 8S-T). Thus, LYPD3 was identified to exert immunosuppressive functions through S100A9-related signaling.

## Discussion

Β-catenins are now considered to be classical downstream targets of Wnt signaling and play a key role in cell–cell junctions [51]. Disturbances in Wnt/β-catenin signaling often lead to tumorigenesis [52, 53]. In most cases, there are several additional catenins that are structurally as well as functionally related to β-catenin, as evidenced in particular by the fact that other catenins are responsive to classical Wnt signaling [54]. There is growing evidence that multiple catenin proteins localized in the nucleus form interactive networks involved in the regulation of gene expression and protein interactions, particularly in the context of actin-based cell morphology and cell motility [55–59].

In the present study, we described the genomic and transcriptomic heterogeneity of 12 catenins at the pan-cancer level and found that the aberrant expression of catenins may be associated with genomic variants, which is consistent with current reports. Interestingly, there are 2 RNA expression patterns of catenins in TCGA-SKCM, where *PKP1*, *JUP*, *PKP3* belong to the same group and are deleterious for survival in melanoma patients. Among them, JUP belongs to the β-catenin superfamily and is structurally similar to β-catenin, while PKP1 and PKP3 belong to the plakophilin branch, which belongs to the largest subfamily, δ-catenins [60]. This seems to suggest a potential heterogeneity of catenins. In TCGA-SKCM, we found that the collective up-regulation of *JUP*, *PKP1*, and *PKP3* could define a distinct catenin subgroup in melanoma (C2), which possesses poorer survival and more aggressive clinical features compared to another subgroup (C1). Previous studies confirmed that PKP1 and PKP3 levels are elevated in several cancer tissues compared to normal tissues and regulate the proliferation and invasive capacity of cancer cells. Our study suggests that imbalanced expression of catenins correlates with the malignant features of melanoma.

Transcriptional differences between different catenin subtypes have been shown to involve a wide range of biological signals [61, 62]. To mine key regulatory mechanisms, MEGENA was performed to characterize the correlations between genes and genes in the module. Based on the correlation network, we obtained the 10 most significant hub genes, among which *LYPD3* had the highest expression abundance and was therefore identified for study. LYPD3, also known as C4.4A, is a membrane protein that is partially anchored to the cell surface by glycosylphosphatidylinositol (GPI) [63]. LYPD3 has been shown to be highly expressed in several human malignancies and is associated with poor prognosis [37, 38, 64]. Interestingly, LYPD3 expression is markedly attenuated in the non-invasive stage of cutaneous malignant lesions (carcinoma in situ), but re-activated upon transformation to malignant invasive squamous cell carcinoma, especially in the invasive front region. This wonderful phenomenon links LYPD3 to the process of tumor infiltration. In the present study, we found that *LYPD3* levels were accompanied by a progressive increase in the depth of melanoma infiltration and confirmed the statistical correlation between high *LYPD3* level and poor OS and DSS. A study showed that LYPD3 stimulates proliferation, migration, invasion and drug resistance of pancreatic ductal adenocarcinoma (PDAC) cells by interacting with AGR2 [41]. In the present study, we demonstrated that JUP can regulate the level of AGR2/LYPD3 and constructed the JUP/AGR2/LYPD3 signaling axis. It was shown that hyperactivation of the JUP/AGR2/LYPD3 signaling axis contributes to the regulation of melanoma cell stemness and promotes glycolysis through a Glucose Transporter Type 1 (GLUT1)-dependent pathway. In colorectal cancer models, LYPD3 was found to be preferentially expressed in CSCs and to play a role in maintaining CSCs [65]. Notably, the correlation between *JUP*, *AGR2*, and *LYPD3* was significantly stronger in C2 than in C1, which may be able to partially explain the higher stemness score in C2 than in C1.

A central feature of catenins is their association with calreticulin, and the two form protein complexes that directly or indirectly regulate cytoskeletal shaping, which allows them to be extensively involved in cell adhesion, motility, and material transfer [59, 66–68]. In addition, CSCs are morphologically characterized as more invasive. In the present study, we demonstrated that upregulation of JUP/AGR2/LYPD3 signaling promotes melanoma cytoskeleton remodeling and stimulates the formation of new pseudopods.

The expression of immunosuppressive cytokines and inhibitory co-stimulatory molecules in CSCs gives CSCs an immunosuppressive potential [69]. Our data suggest that melanoma cells in an undifferentiated state have weak communication with immune cells. Notably, C2 presented an immunosuppressed state (low level of immune infiltration) and we observed a negative correlation between *LYDP3* level and CD8 + T cells only in C2, but not in C1. These results imply a link between LYPD3 and immunosuppressive TME. Subsequent analysis showed that LYPD3 was co-expressed on myeloid cells with S100A9, which has been shown to mediate dysfunction of effector T cells thereby affecting the response to immunotherapy. Thus, LYPD3 is a potential immunosuppressive factor.

This study extends the biological significance of the JUP/AGR2/LYPD3 signaling axis to melanoma. The development of an anti-tumour drug (bay 1129980) targeting C4.4A is currently underway and has shown effective therapeutic effects in a human-derived tumour xenograft model [36]. We hope to provide some basis for expanding the clinical applications of this drug. We recognise that our study has some limitations. The clinical studies included in this study are based on public databases and may be biased. We are collecting patients in a multicentre clinical cohort to further analyse and validate the significance of LYPD3 in immunotherapy, and large-scale sequencing analyses are necessary. Our findings suggest that the JUP/AGR2/LYPD3 signaling axis plays an important role in melanoma, but its in-depth mechanism of action is still lacking. Our team is conducting further research on this topic.

## Conclusion

The JUP/AGR2/LYPD3 signaling axis plays an important role in the malignant features of melanoma. Targeting the JUP/AGR2/LYPD3 signaling axis can help develop promising drugs.

### Supplementary Information

**Additional file 1**. Supplementary information.

## Data Availability

The data and materials in the current study are available from the corresponding author: Song-yang Xi: 20215011@njucm.edu.cn.
