# Supplementary material for: LY6/PLAUR domain containing 3 (LYPD3) maintains melanoma cell stemness and mediates an immunosuppressive microenvironment
Source: Biol Direct. 2023 Nov 3;18:72. doi: 10.1186/s13062-023-00424-3 (PMC10623712; doi:10.1186/s13062-023-00424-3)
Supplement: Supplementary file 1 — Additional file 1. Supplementary information. [file 13062_2023_424_MOESM1_ESM.docx]

**Supplementary Table 1**

| **Antibodies and Reagents** | **Manufacturer, Country, Cat number** | **Concentration** |
| --- | --- | --- |
| Dulbecco’s modified Eagle’s medium (DMEM) | Gibco, USA, #8121032 |  |
| Fetal bovine serum (FBS) | Gibco, USA, # 42F1376K |  |
| AGR2 Rabbit Antibody | Cell Signaling Technology, USA, #13062 | WB: 1:1000 |
| C4.4A/LYPD3 Rabbit Monoclonal Antibody | Beyotime Biotechnology, China, # AG4939 | WB: 1:1000  IF: 1:50 |
| Glut1 (E4S6I) Rabbit Antibody | Cell Signaling Technology, USA, #73015 | WB: 1:1000 |
| GAPDH (D16H11) Rabbit Antibody | Cell Signaling Technology, USA, #5174 | WB: 1:10000 |
| Anti-β-actin Antibody | Proteintech, China, # 81115-1-RR | WB: 1:5000 |
| Anti-F-actin | Abcam, UK, #ab130935 | IF: 1:500 |
| HE staining kit | Beyotime Biotechnology, China, #C0105, | - |
| Goat Anti-Rabbit IgG H&L (HRP) | Abcam, UK, #ab205718 | IHC 1:2000 |
| Goat anti-mouse IgG (H+L) CoraLite594 | Proteintech, China, #SA00013-4 | IF: 1:500 |
| Fluorescein (FITC) conjugated Affinipure Goat Anti-Rabbit IgG(H+L) | Proteintech, China, # SA00003-2 | IF: 1:500 |
| Highly Cross-Adsorbed Goat (Polyclonal) Anti-Mouse IgG(H+L) Antibody | LI-COR, USA, 926-68070, Q04695 | WB 1:5000 |
| Highly Cross-Adsorbed Goat (Polyclonal) Anti-Rabbit IgG(H+L) Antibody | LI-COR, USA, 926-68071, S11385 | WB 1:5000 |
| Alexa Fluor®594-conjugated phalloidin | Solarbio, China, # CA1680 | - |
| Bicinchoninic acid (BCA)  Assay Kit | Thermo Fisher Scientific, USA, #23227 |  |
| Recombinant Human EGF  Animal-Free manufactured | Peprotech, USA, # AF-100-15 | 20ng/ml |
| Recombinant Human FGF-basic (154 a.a.) | Peprotech, USA, #100-18B-100 | 20ng/ml |
| B27 supplement | Gibco, USA, #17504044 | 2% |
| Mouse glucose transporter 1 (GLUT1) ELISA kit | BIOESN, China, #BES5142K |  |
| Lentiviral vectors of JUP | GeneChem, China, #GIEL0304604 |  |
| Lentiviral vectors of LYPD3 | GeneChem, China, #GIEE0307722 |  |
| Puromycin | Beyotime Biotechnology, China, # ST551 | 1.5mg/ml |
| Geneticin | Beyotime Biotechnology, China, # ST081 | 1.0mg/ml |
| 0.2% TritonX-100 | Proteintech, China, #61202011 |  |
| Nuclei were stained using 4',6-diamidino-2-phenylindole (DAPI) | Beyotime Biotechnology, China, # 091620210520 |  |

**Supplementary Methods**

**Melanoma-Cohort (GEO) batch effect correction**

The independent validation cohort (bulk data) contains three datasets (GSE22153, GSE59455, and GSE65904) from the GEO database and is labelled as a merged GEO (mGEO) cohort[1, 2]. The “Combat” algorithm in the SVA package was used to correct for batch effects in the GEO cohort.

**Construction of catenin phenotypes**

We downloaded data on 10 catenin molecules from HUGO Gene Nomenclature Committee (HGNC) (http://www.genenames.org/): *CTNNA1*, *CTNNA2*, *CTNNA3*, *CTNNB1*, *JUP*, *PKP1*, *PKP2*, *PKP3*, *ARVCF*, *CTNND1*, *CTNND2*, and *PKP4*[3]. We first conducted unsupervised clustering analysis based on the transcriptional profiles of 10 catenin molecules to identify the catenin phenotypes in melanoma, so as for stratification of patients. The optimal clustering number of SKCM-Cohort was determined by the multiple criteria within consensus clustering algorithm, and its repeatability was verified in a independent cohort (mGEO). The clustering process was performed through R package “ConsensusClusterPlus”[4].

**Single-Cell Analysis**

Single-cell RNA sequencing (scRNA-seq) data GSE189889 were downloaded from the GEO database[5]. All data were incorporated into Seurat V4 and filtered according to the criteria of the authors of the data sources, and the filtered cells were analysed by uniform surface approximation and projection (UMAP) and t-distributed stochastic neighbourhood embedding (t-SNE)[6]. Melanoma cells were then identified from all cells by automatic annotation. The "FindAllMarkers" function (min.pct=0.25, logfc.threshold=1, tes.use="Wilcox") was called to evaluate marker genes for each cell subpopulation[7]. The "Dotplot" and "Vlnplot" functions were used to visualise the results. The "Cellchat" package was used to infer interactions between cell subpopulations[8]. In addition, the "hdWGCNA" package was used to identify gene modules specific to particular cell subpopulations[9].

To assess differences in characteristics between different cellular subpopulations, we used “CytoTRACE” package to further analyse the scRNA-seq data to infer cellular differentiation status[10], and “scMetabolism” package to assess single-cell metabolic activation scores[11].

**Enrichment analysis**

To analyze the enrichment of Gene Ontology (GO) and Kyoto Encyclopedia of Genomes (KEGG), the ClusterProfiler toolkit was used[12]. In addition, the Metascape web tool was conducted to compare specifically enriched gene sets between different gene modules.

**Immune Analysis**

We calculated the abundance of immune cell infiltration in TCGA-SKCM samples using the Tumour Immunity Estimation Resource (TIMER)[13], QUANTISEQ[14], ssGSEA and EPIC algorithms built into the “Immuno-Oncology-Biological-Research (IOBR) software package[15, 16].

**Construction of Co-Expression Networks**

The R software package “multiscale embedded gene co-expression network analysis (MEGENA)” was applied to distinguish the co-expression network[17]. The specific calculation process is as described in the previous studies[18]. In addition, we ranked the genes within the module by degree value to identify important hub genes.

**Identification of differentially expressed genes (DEGs)**

The R package “Limma” was used to identify DEGs between two patterns of catenin modification. the significance criterion was set at P < 0.05[19].

**Cell Culture**

The A375 and SK-MEL-28 malignant melanoma cell line were obtained from the Wuhan Pu-nuo-sai Life Technology Co. Ltd. (Wuhan, China) and cultured in Dulbecco's modified Eagle's medium (DMEM) supplemented with 10% fetal bovine serum (FBS) and 100 U/ml penicillin/100 µg/ml streptomycin at 37 °C with 5% CO_2_.

## Western blotting

Cells were lysed in radioimmunoprecipitation (RIPA) buffer and protein concentrations measured by the Bradford assay[20]. Samples of 20 µg each were separated on 10% or 8% SDS–polyacrylamide gel electrophoresis (SDS–PAGE). Proteins were transferred to polyvinylidene fluoride (PVDF) membranes and blocked with 5% bovine serum albumin. The blots were then probed with the relevant primary antibodies at 4˚C overnight. After washing three times in Tris-buffered saline containing 0.05% Tween-20, the blots were incubated with the corresponding secondary antibodies and an Electrochemiluminescence (ECL) detection kit used to measure densities. The GAPDH/β-actin protein was used as a reference.

## Transfection

Each lentiviral and Plasmid vector, containing sh-JUP, JUP overexpression plasmid (oe-JUP), sh-LYPD3, and the corresponding empty vector (NC), was constructed, sequenced, packaged as a virus, and tested for titer by Genechem. The transfection procedure was carried out in accordance with the protocol provided by the manufacturer. For 24 hours, 2 μg/mL polybrene transduced A735 and SK-MEL-28 cells with the lentivirus. Following that, transduced cells were selected with 1.5 μg/mL puromycin or 1.0 μg/mL Geneticin. Western blots were used to evaluate the transduction efficiency.

## Xenograft tumor model

The ethical committee of the Zhangjiagang TCM Hospital Affiliated to Nanjing University of Chinese Medicine granted approval for all animal experiments conducted (approval number: 2022-10-012). All experiments were performed according to ARRIVE2.0 guidelines[21]. A total of 30 male BALB/c nude mice, aged 4 weeks, were acquired from the Beijing Weitong Lihua Experimental Animal Technology Co., Ltd (Certificate No. SYXK2019-0010). Nude mice weighed in the range of 18-22 g and were fed under specific pathogen-free conditions.SK-MEL-28 cells (4×10^6^ cells/mouse) were subcutaneously injected into the right armpit region of the mice (n = 6 per group) and were transfected with sh-JUP, oe-JUP, NC, si-LYPD3, and control cells. Transfected cells were subcutaneously injected into BALB/c nude mice. Subcutaneous tumor formation was observed seven days later. The maximum (a) and minimum tumor diameter (b) were assessed biweekly. On the 28th day of the experiment, the mice were subjected to euthanasia, and subsequently, all tumors were gathered for analysis. The volume of the tumor was estimated (V = 1/2ab2), and the growth curves of the subcutaneous xenografts were drawn.

## Colony-formation and spheroid-formation assay

The colony-formation assays were performed in accordance with the established methodology. In this study, A735 or SK-MEL-28 cells were seeded in six-well plates at a density of 1 × 10^3^ cells per well. The medium was replenished at a frequency of once every three days. Crystal violet staining was done after two weeks. The quantification of colonies was conducted using a compound light microscope (Olympus BX53, Japan). Tumor spheroids were generated using the methodology previously outlined. Briefly, ultralow adhesion six-well plates were seeded with 1 × 10^4^ single-cell suspensions in 37 °C incubators. After 2 weeks of culture in defined media with 1% B27, 20 ng/ml human EGF, and 20 ng/ml human FGF, cell spheres were imaged and counted under ×200 magnification (Olympus BX53, Japan).

## Hematoxylin and eosin (HE) staining

We employed a kit and associated directions for HE staining. In short, following generation of 4-μm-thick tissue section, paraffin-embedding, fixation in 4% paraformaldehyde, a 1-min treatment with 5% acetic acid, 1 min de-waxing, ethanol hydration using a series of concentrations, 5 min hematoxylin staining at room temperature, 1 min eosin staining, routine dehydration, clearance, and sealing, pathological alterations within sections were assessed under an Olympus IX81 microscope (Japan).

## Ethics and sample collection

The use of specimens was carried out with the informed consent of the patients in accordance with the principles of the Declaration of Helsinki This work received ethical approval from Zhangjiagang TCM Hospital Affiliated to Nanjing University of Chinese Medicine (approval number: 2022-03-14). Patient data was randomized and anonymized before analyses. Frozen samples were acquired, and maintained in liquid nitrogen. All patient records/information were anonymized and de-identified prior to analysis. Each sample was collected frozen and immediately stored in liquid nitrogen. Inclusion criteria:(1) All patients were first pathologically diagnosed with SKCM. (2) The diagnosis was referenced to the latest (8th edition) American Joint Committee on Cancer (AJCC) SKCM staging system[22]. (3) Patients with SKCM who were hospitalized in the Zhangjiagang TCM Hospital Affiliated to Nanjing University of Chinese Medicine from 2022-05-01 to 2023-05-01. Also, pathological specimens from each melanoma patient were kept for independent re-diagnostic confirmation by two doctors. (4) All patients had complete clinical information. Exclusion criteria: (1) Patients with a clinical diagnosis of a tumor that has metastasised to the skin from another site. (2) Exclusion of any other serious chronic disease or other visceral tumors. (3) Incomplete clinical data. Finally, a total of 30 patients with SKCM were analyzed.

## Immunohistochemical (IHC) and Immunofluorescence (IF) staining

The protocols for IHC and IF staining’s were adopted from previous investigations[23], and the employed antibodies are presented in Supplemental Table 1. Antibody final concentrations were either according to prior research or provider recommendations. Following tissue section blocking with protein blocking solution, slides underwent incubation in primary antibody. The IHC scoring (staining intensity and extent) was conducted by two independent researchers. Image capture was done using a NIKON Eclipse Ni-E microscope (NIKON, Japan), and the stain grading was performed as follows: 0, negative; 1, weak; 2, moderate; and 3, strong. The staining extent was graded dependent on the amount of positive vs. negative cells in each specimen: 0, no positive staining; 1 <10% positive staining; 2, 10–50% positive staining; and 3, >50% positive staining. The IHC score (H-SCORE) integrating the amount of stained cells and staining intensity, was computed as follows: H-SCORE= ∑(PI × I) = (percentage of cells with weak intensity × 1) + (percentage of cells with moderate intensity × 2) + (percentage of cells with strong intensity × 3). Herein, PI referred to the percentage of positive cells to the entire quantity of cells in a given field of view, and I represented staining intensity. The H-SCORE ranged between 0-300, with a higher score denoting enhanced positive staining. Tissue sections were blocked with either 5% bovine serum albumin in phosphate-buffered saline (PBS) or in 0.1% Tween, prior to a overnight treatment in 4 °C with primary antibody, and subsequent treatment with secondary antibodies in 25 °C for 1hour. A 3-min nuclear staining was conducted without light with 4',6-diamidino-2-phenylindole (DAPI). Then, sections underwent a 5-min PBS-rinse four separate times, followed by sealing with a solution containing a IF quencher. IF staining observation and image capture was done via an inverted IF Olympus CKX-41 microscope (Japan).

## Construction of JUP lentiviral vectors

**Supplementary Table 2. (Gene information):**

| **Gene symbol** | **GenBank_ID** |
| --- | --- |
| JUP | NM_001352773.2 |

**Supplementary Table 3 (Easy-siRNA design):**

| **NO.** | **Accession** | **Target Seq** | **CDS** | **GC%** |
| --- | --- | --- | --- | --- |
| JUP-RNAi (102467-1) | NM_001352773.2 | gcATGCTATCACATGTCACAT | 80..2215 | 42.11% |

**Plasmid name：**GV493

**Element’s order：**hU6-MCS-CBh-IRES-puromycin

**Negative control number：**CON313

**Negative control insert sequence：**ATCTCCGAAGCTGTCCATG

**Overexpression Plasmid information：**

**Plasmid name：**GV492

**Element’s order：**Ubi-MCS-3FLAG-CBh -IRES-puromycin

**The cloning site:** BamHI / AgeI

**Control number：**CON335

## Construction of LYPD3 lentiviral vectors

**Supplementary Table 4 (Gene information):**

| **Gene symbol** | **GenBank_ID** |
| --- | --- |
| LYPD3 | NM_014400.3 |

**Table S6 (Target information)**

| **ID** | **Target Sequence information：** | **Starting position information** | **GC content (%)** |
| --- | --- | --- | --- |
| LYPD3-RNAi (54531-1) | cgATAGTTGCTGGCTACAATC | 262 | 36.84 |

**Plasmid name：**GV493

**Element’s order：**hU6-MCS-CBh-IRES-geneticin

**Negative control number：**CON313

**Negative control insert sequence：**TTCTCCGAACGTGTCACGT

**Supplementary Figure**


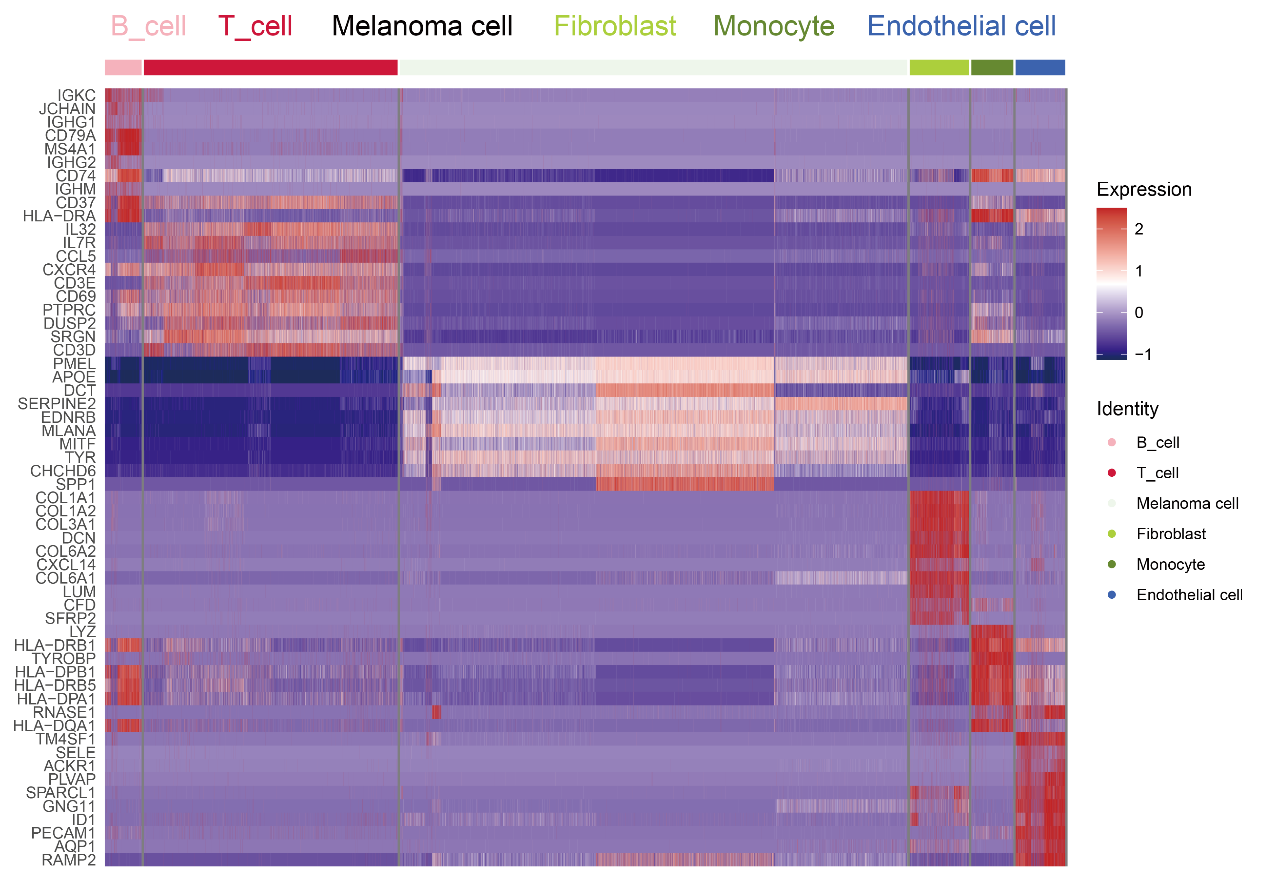


Figure S1. Heatmap showing the relative expression level of genes across six cell types (cell-type markers). Cell type markers were identified by unbiased estimation (Wilcoxon rank sum test, FDR < 0.01, fold change > 1.5), and only the top ten are shown in the figure.


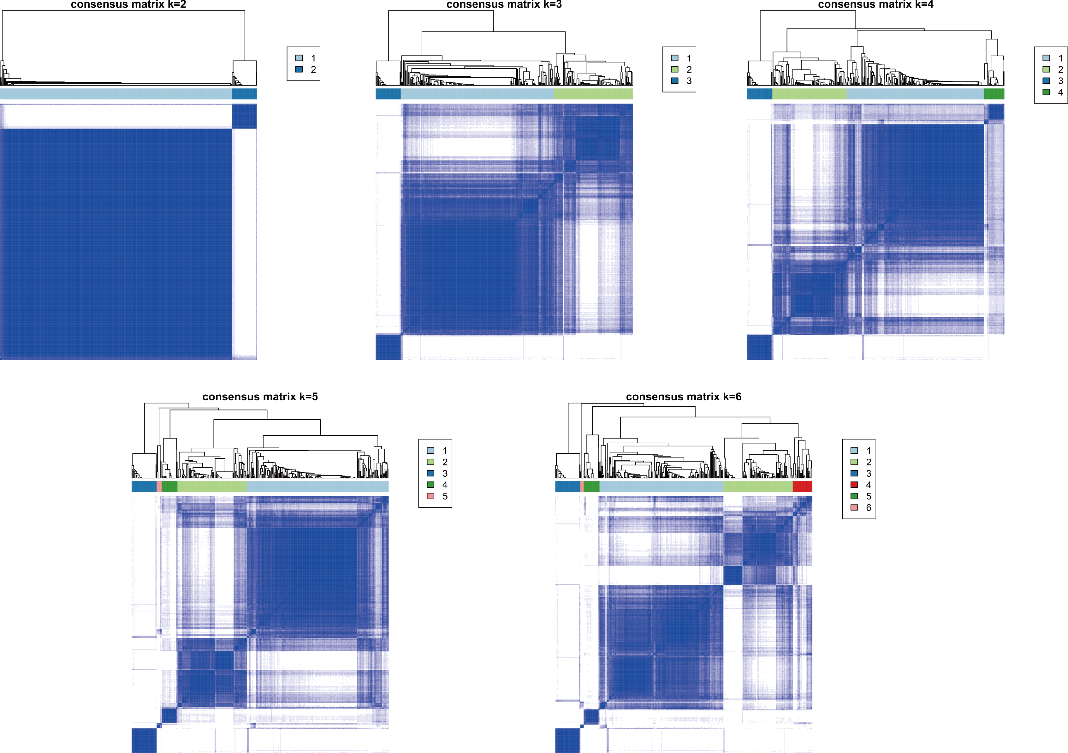


Figure S2. The optimal number of clusters was determined to be two by R package “ConsensusClusterPlus”.


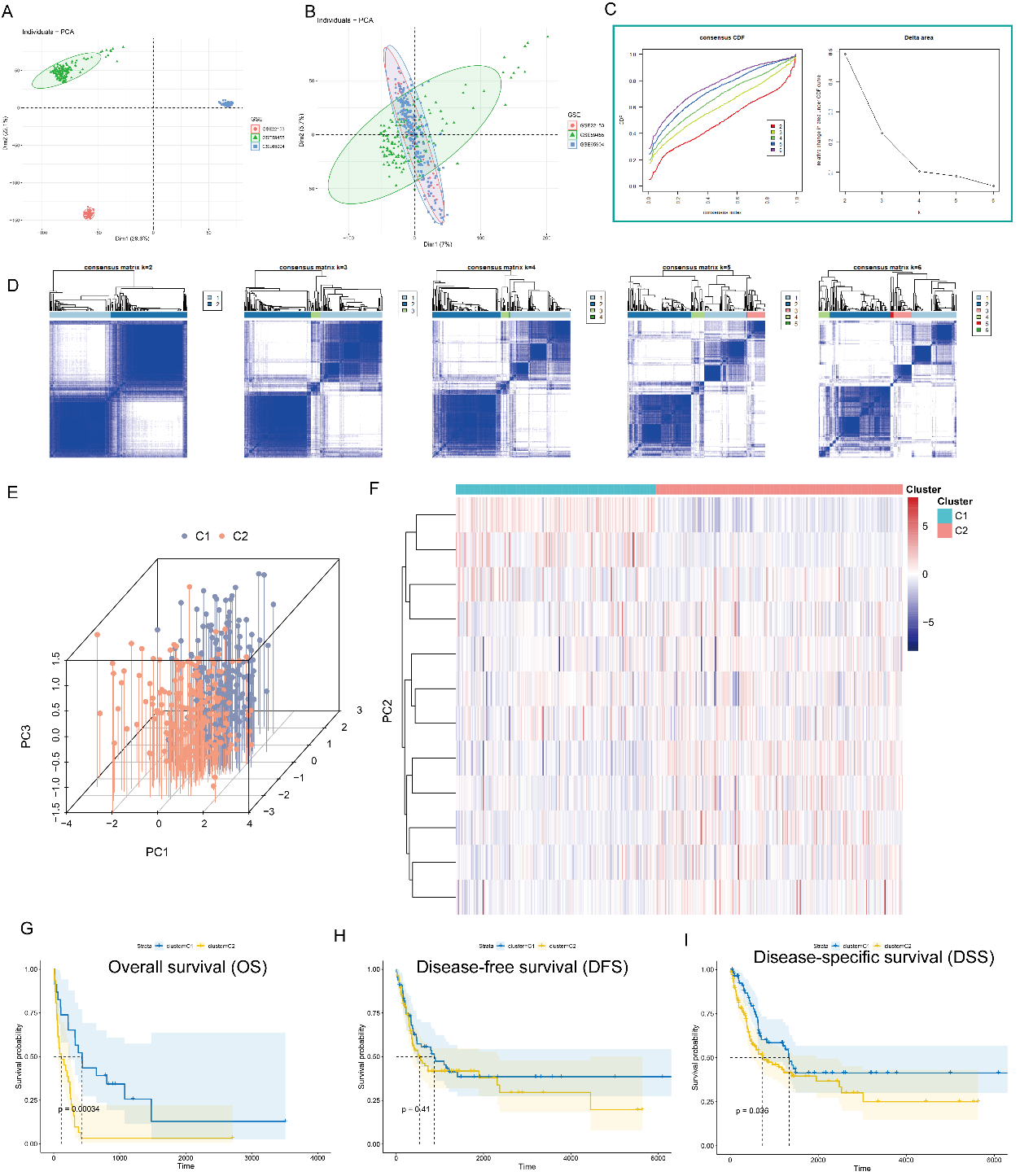


Figure S3. Unsupervised clustering to obtain two different subtypes of catenin in merged GEO (mGEO) dataset. (A-B) Principal component analysis (PCA) showing the gene expression distribution in 3 GEO melanoma cohort samples (GSE22153, GSE59455, and GSE65904) before (A) and after (B) batch effect correction. (C) Left: The cumulative distribution function (CDF) curves in consensus cluster analysis. CDF curves of consensus scores by different subtype numbers (k = 2, 3, 4, 5, and 6) were displayed. Right: Relative change in area under the CDF curve for k = 2–6. (D) The consensus score matrix of melanoma cases in mGEO when k = 2. The higher the consensus score was, the more likely they were assigned to the same group. (E) Three-dimensional principal component analysis plot showing the distribution of melanoma examples based on catenin levels when k=2. Each point represents a sample and different sample clusters are marked using different colors. (F) Heatmap showing differences in expression of 10 catenin molecules between two melanoma clusters. (G-I) Overall survival (OS) (G), Disease-free survival (DFS) (H), and Disease specific survival (DSS) (I) analysis to the catenin subtype was performed. Log rank test was conducted.


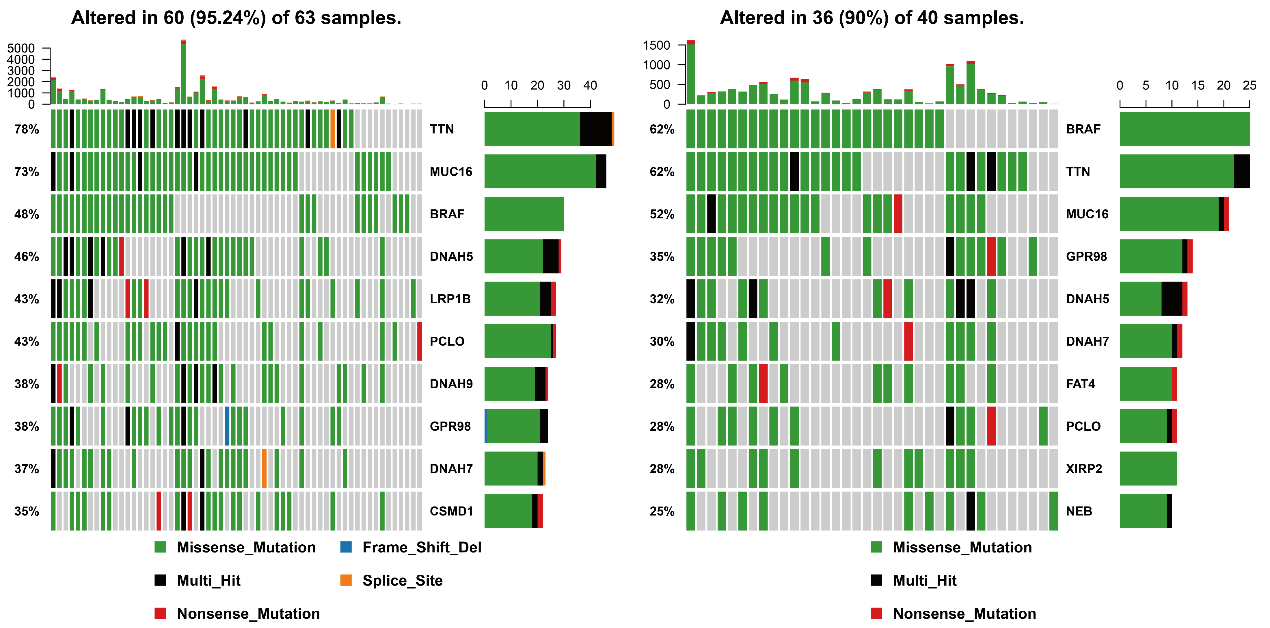


Figure S4. Oncoplot showing mutations in the top 10 genes union set with the highest mutation frequency in C1 (left) and C2 (right), respeectively.


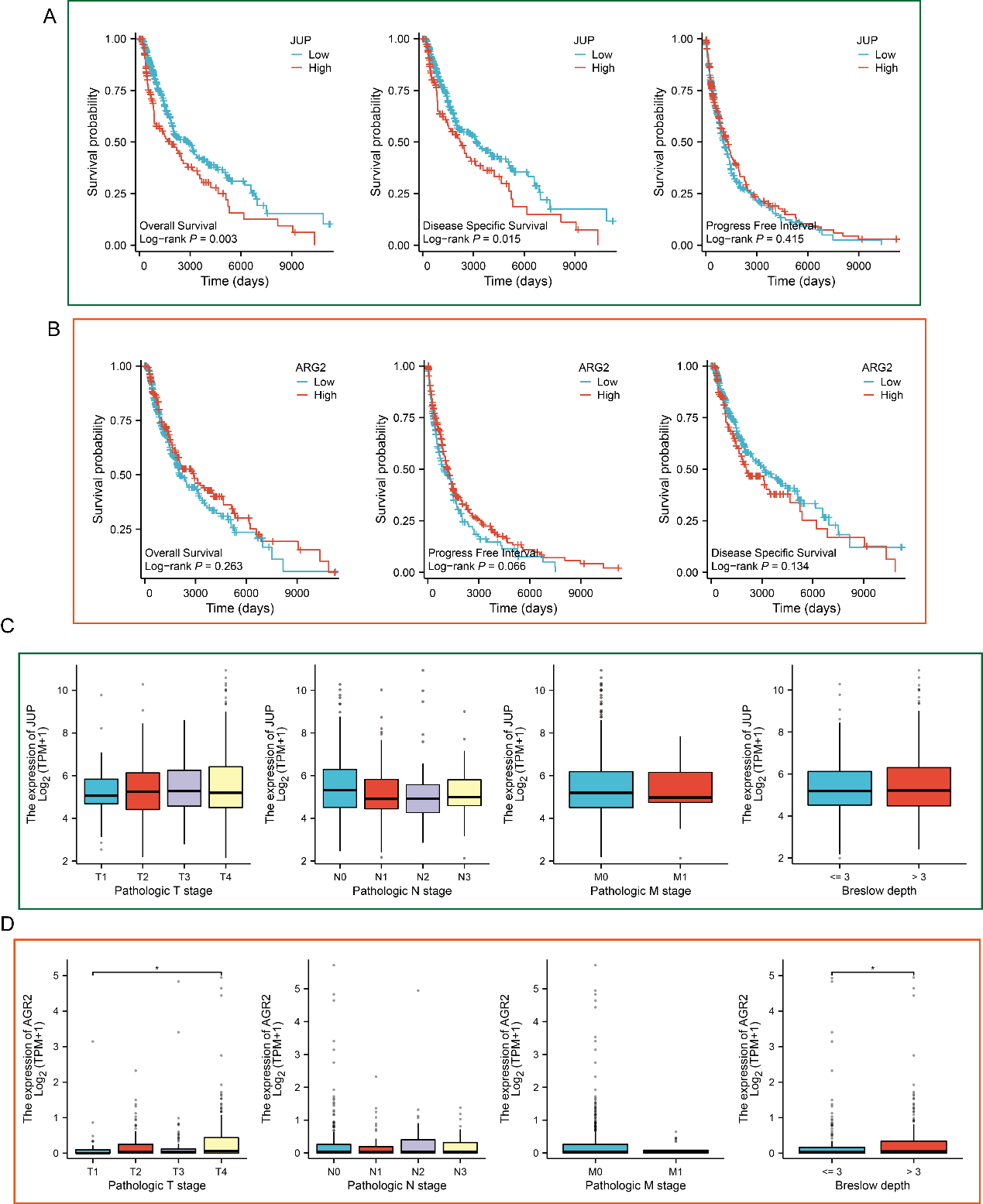


Figure S5. Correlation between AGR2, JUP levels and clinical profile of melanoma patients. (A-B) The expression level of JUP (A) and AGR2 (B) among different clinical stages (T, N, M stage, and Breslow depth) was analyzed in TCGA-SKCM. (C-D) OS, DSS, and Progress Free Interval (PFI) analysis according to the expression level of the *JUP* (C) and *AGR2* (D) gene were performed using melanoma cases in the TCGA-SKCM cohort.


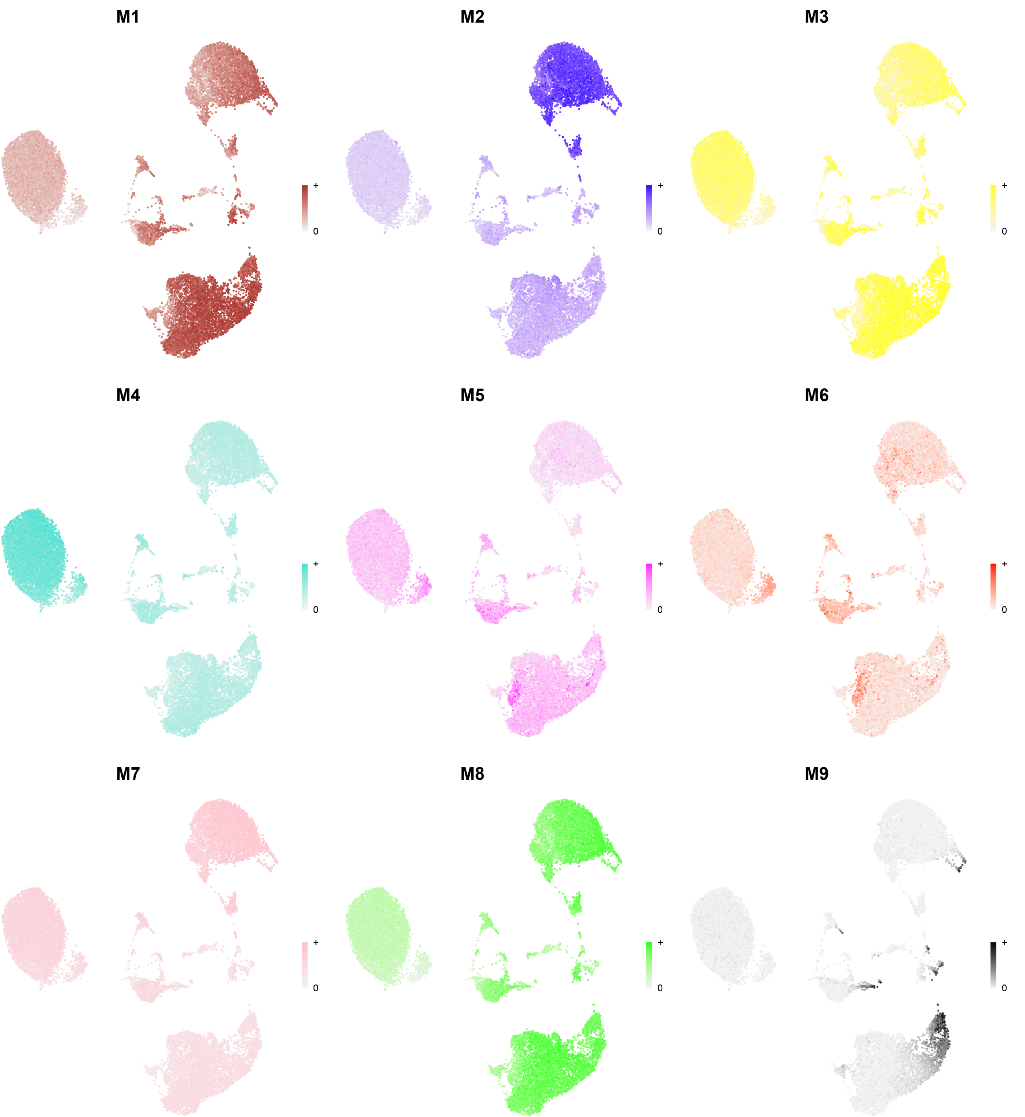


Figure S6. Distribution of gene module scores in melanoma cells. There are total ninne gene modules for melanoma cells.

**References**

1. Liu, Y., et al., *Construction and Validation of a Ferroptosis-Related Prognostic Signature for Melanoma Based on Single-Cell RNA Sequencing.* Front Cell Dev Biol, 2022. **10**: p. 818457.

2. Yan, K., et al., *9-Gene Signature Correlated With CD8(+) T Cell Infiltration Activated by IFN-γ: A Biomarker of Immune Checkpoint Therapy Response in Melanoma.* Front Immunol, 2021. **12**: p. 622563.

3. Gray, K.A., et al., *A review of the new HGNC gene family resource.* Hum Genomics, 2016. **10**: p. 6.

4. Wilkerson, M.D. and D.N. Hayes, *ConsensusClusterPlus: a class discovery tool with confidence assessments and item tracking.* Bioinformatics, 2010. **26**(12): p. 1572-3.

5. *Single cell characterization of the cellular landscape of acral melanoma identifies novel targets for immunotherapy*.

6. Voigt, A.L., et al., *Metabolic transitions define spermatogonial stem cell maturation.* Hum Reprod, 2022. **37**(9): p. 2095-2112.

7. Hu, J., et al., *Development of a Cancer-Associated Fibroblast-Related Prognostic Model in Breast Cancer via Bulk and Single-Cell RNA Sequencing.* Biomed Res Int, 2022. **2022**: p. 2955359.

8. Chen, B., et al., *A Cuproptosis Activation Scoring model predicts neoplasm-immunity interactions and personalized treatments in glioma.* Comput Biol Med, 2022. **148**: p. 105924.

9. Morabito, S., et al., *hdWGCNA identifies co-expression networks in high-dimensional transcriptomics data.* Cell Rep Methods, 2023. **3**(6): p. 100498.

10. Senra, D., N. Guisoni, and L. Diambra, *ORIGINS: A protein network-based approach to quantify cell pluripotency from scRNA-seq data.* MethodsX, 2022. **9**: p. 101778.

11. Wu, Y., et al., *Spatiotemporal Immune Landscape of Colorectal Cancer Liver Metastasis at Single-Cell Level.* Cancer Discov, 2022. **12**(1): p. 134-153.

12. Zhao, X., et al., *Identification of key biomarkers and immune infiltration in systemic lupus erythematosus by integrated bioinformatics analysis.* J Transl Med, 2021. **19**(1): p. 35.

13. Li, T., et al., *TIMER: A Web Server for Comprehensive Analysis of Tumor-Infiltrating Immune Cells.* Cancer Res, 2017. **77**(21): p. e108-e110.

14. Finotello, F., et al., *Molecular and pharmacological modulators of the tumor immune contexture revealed by deconvolution of RNA-seq data.* Genome Med, 2019. **11**(1): p. 34.

15. Lu, H., et al., *Identifying a Novel Defined Pyroptosis-Associated Long Noncoding RNA Signature Contributes to Predicting Prognosis and Tumor Microenvironment of Bladder Cancer.* Front Immunol, 2022. **13**: p. 803355.

16. Luo, D., et al., *Holliday Cross-Recognition Protein HJURP: Association With the Tumor Microenvironment in Hepatocellular Carcinoma and With Patient Prognosis.* Pathol Oncol Res, 2022. **28**: p. 1610506.

17. Song, W.M. and B. Zhang, *Multiscale Embedded Gene Co-expression Network Analysis.* PLoS Comput Biol, 2015. **11**(11): p. e1004574.

18. Yin, Y., et al., *Identification and Validation in a Novel Classification of Helicase Patterns for the Prediction of Tumor Proliferation and Prognosis.* J Hepatocell Carcinoma, 2022. **9**: p. 885-900.

19. Ritchie, M.E., et al., *limma powers differential expression analyses for RNA-sequencing and microarray studies.* Nucleic Acids Res, 2015. **43**(7): p. e47.

20. Sapan, C.V., R.L. Lundblad, and N.C. Price, *Colorimetric protein assay techniques.* Biotechnol Appl Biochem, 1999. **29**(2): p. 99-108.

21. Percie du Sert, N., et al., *Reporting animal research: Explanation and elaboration for the ARRIVE guidelines 2.0.* PLoS Biol, 2020. **18**(7): p. e3000411.

22. Scolyer, R.A., et al., *Melanoma pathology reporting and staging.* Mod Pathol, 2020. **33**(Suppl 1): p. 15-24.

23. Liu, Y.J., et al., *FSTL3 is a Prognostic Biomarker in Gastric Cancer and is Correlated with M2 Macrophage Infiltration.* Onco Targets Ther, 2021. **14**: p. 4099-4117.
